# The Relationship Between Polygenic Risk Scores and Cognition in Schizophrenia

**DOI:** 10.1093/schbul/sbz061

**Published:** 2019-06-17

**Authors:** Alexander L Richards, Antonio F Pardiñas, Aura Frizzati, Katherine E Tansey, Amy J Lynham, Peter Holmans, Sophie E Legge, Jeanne E Savage, Ingrid Agartz, Ole A Andreassen, Gabriella A M Blokland, Aiden Corvin, Donna Cosgrove, Franziska Degenhardt, Srdjan Djurovic, Thomas Espeseth, Laura Ferraro, Charlotte Gayer-Anderson, Ina Giegling, Neeltje E van Haren, Annette M Hartmann, John J Hubert, Erik G Jönsson, Bettina Konte, Leonhard Lennertz, Loes M Olde Loohuis, Ingrid Melle, Craig Morgan, Derek W Morris, Robin M Murray, Håkan Nyman, Roel A Ophoff, GROUP Investigators, Jim van Os, EUGEI WP2 Group, Schizophrenia Working Group of the Psychiatric Genomics Consortium, Tracey L Petryshen, Diego Quattrone, Marcella Rietschel, Dan Rujescu, Bart P F Rutten, Fabian Streit, Jana Strohmaier, Patrick F Sullivan, Kjetil Sundet, Michael Wagner, Valentina Escott-Price, Michael J Owen, Gary Donohoe, Michael C O’Donovan, James T R Walters

**Affiliations:** 1 MRC Centre for Neuropsychiatric Genetics and Genomics, Division of Psychological Medicine and Clinical Neurosciences, School of Medicine, Cardiff University, Cardiff, UK; 2 Complex Trait Genetics Lab, Center for Neurogenomics and Cognitive Research, Vrije Universiteit Amsterdam, Amsterdam, The Netherlands; 3 Norwegian Centre for Mental Disorders Research, KG Jebsen Centre for Psychosis Research, Division of Mental Health and Addiction, Oslo University Hospital and Institute of Clinical Medicine, University of Oslo, Oslo, Norway; 4 Department of Psychiatric Research, Diakonhjemmet Hospital, Oslo, Norway; 5 Centre for Psychiatry Research, Department of Clinical Neuroscience, Karolinska Institutet and Stockholm Health Care Services, Stockholm County Council, Stockholm, Sweden; 6 CoE NORMENT, KG Jebsen Centre for Psychosis Research, Division of Mental Health and Addiction, Oslo University Hospital and Institute of Clinical Medicine, University of Oslo, Oslo, Norway; 7 Psychiatric and Neurodevelopmental Genetics Unit, Center for Genomic Medicine, Massachusetts General Hospital, Boston, MA; 8 Department of Psychiatry and Neuropsychology, School for Mental Health and Neuroscience, Faculty of Health, Medicine and Life Sciences, Maastricht University, Maastricht, The Netherlands; 9 Department of Psychiatry, Harvard Medical School, Boston, MA; 10 Stanley Center for Psychiatric Research, Broad Institute of MIT and Harvard, Cambridge, MA; 11 Neuropsychiatric Genetics Research Group, Department of Psychiatry, Institute of Molecular Medicine, Trinity College Dublin, Dublin, Ireland; 12 Cognitive Genetics and Cognitive Therapy Group, Neuroimaging and Cognitive Genomics Center, School of Psychology and Discipline of Biochemistry, National University of Ireland Galway, Galway, Ireland; 13 Department of Genomics, Life and Brain Center, University of Bonn, Bonn, Germany; 14 Institute of Human Genetics, University of Bonn, Bonn, Germany; 15 Department of Medical Genetics, Oslo University Hospital, Oslo, Norway; 16 Department of Experimental Biomedicine and Clinical Neuroscience, University of Palermo, Palermo, Italy; 17 Department of Health Service and Population Research, Institute of Psychiatry, King’s College London, London, UK; 18 Department of Psychiatry, Psychotherapy and Psychosomatics, Martin-Luther-University Halle-Wittenberg, Halle, Germany; 19 Department of Psychiatry, Brain Center Rudolf Magnus, University Medical Center Utrecht, Utrecht University, Utrecht, The Netherlands; 20 Department of Child and Adolescent Psychiatry/Psychology, Sophia Children’s Hospital, Erasmus Medical Center, Rotterdam, The Netherlands; 21 Department of Psychiatry and Psychotherapy, University of Bonn, Bonn, Germany; 22 Center for Neurobehavioral Genetics, Semel Institute for Neuroscience and Human Behavior, University of California, Los Angeles, Los Angeles, CA; 23 Division of Mental Health and Addiction, Oslo University Hospital, Oslo, Norway; 24 National Institute for Health Research (NIHR) Mental Health Biomedical Research Centre at South London and Maudsley NHS Foundation Trust and King’s College, London, UK; 25 Centre for Neuroimaging and Cognitive Genomics, National University of Ireland Galway, Galway, Ireland; 26 Institute of Psychiatry, Psychology and Neuroscience, King’s College London, London, UK; 27 Department of Human Genetics, David Geffen School of Medicine, University of California, Los Angeles, Los Angeles, CA; 28 Department of Psychiatry and Medical Psychology, Maastricht University Medical Center (MUMC+), Maastricht, The Netherlands; 29 Department of Psychiatry, Utrecht University Medical Centre, Utrecht, The Netherlands; 30 King’s Health Partners Department of Psychosis Studies, King’s College London, Institute of Psychiatry, London, UK; 31 Center for Genomic Medicine, Massachusetts General Hospital, Boston, MA; 32 Social, Genetics and Developmental Psychiatry Centre, Institute of Psychiatry, Psychology and Neuroscience, King’s College London, London, UK; 33 Department of Genetic Epidemiology in Psychiatry, Central Institute of Mental Health, Medical Faculty Mannheim/Heidelberg University, Mannheim, Germany; 34 Department of Psychiatry and Neuropsychology, School for Mental Health and Neuroscience, South Limburg Mental Health Research and Teaching Network, Maastricht University Medical Centre, Maastricht, The Netherlands; 35 Central Institute of Mental Health, University of Heidelberg, Medical Faculty Mannheim, Mannheim, Germany; 36 Department of Genetics, University of North Carolina at Chapel Hill, Chapel Hill, NC; 37 Department for Neurodegenerative Diseases and Geriatric Psychiatry, University Hospital Bonn, Bonn, Germany; 38 German Center for Neurodegenerative Diseases (DZNE), Bonn, Germany

**Keywords:** psychiatry, genomics, intelligence, bioinformatics

## Abstract

**Background:**

Cognitive impairment is a clinically important feature of schizophrenia. Polygenic risk score (PRS) methods have demonstrated genetic overlap between schizophrenia, bipolar disorder (BD), major depressive disorder (MDD), educational attainment (EA), and IQ, but very few studies have examined associations between these PRS and cognitive phenotypes within schizophrenia cases.

**Methods:**

We combined genetic and cognitive data in 3034 schizophrenia cases from 11 samples using the general intelligence factor *g* as the primary measure of cognition. We used linear regression to examine the association between cognition and PRS for EA, IQ, schizophrenia, BD, and MDD. The results were then meta-analyzed across all samples. A genome-wide association studies (GWAS) of cognition was conducted in schizophrenia cases.

**Results:**

PRS for both population IQ (*P* = 4.39 × 10^–28^) and EA (*P* = 1.27 × 10^–26^) were positively correlated with cognition in those with schizophrenia. In contrast, there was no association between cognition in schizophrenia cases and PRS for schizophrenia (*P* = .39), BD (*P* = .51), or MDD (*P* = .49). No individual variant approached genome-wide significance in the GWAS.

**Conclusions:**

Cognition in schizophrenia cases is more strongly associated with PRS that index cognitive traits in the general population than PRS for neuropsychiatric disorders. This suggests the mechanisms of cognitive variation within schizophrenia are at least partly independent from those that predispose to schizophrenia diagnosis itself. Our findings indicate that this cognitive variation arises at least in part due to genetic factors shared with cognitive performance in populations and is not solely due to illness or treatment-related factors, although our findings are consistent with important contributions from these factors.

## Introduction

Schizophrenia is an often debilitating, highly heritable mental disorder affecting around 1% of the population.^1^ Individuals with schizophrenia show marked cognitive deficits, on average, compared with healthy controls.^2^ Cognitive impairments are strongly associated with functional outcomes in schizophrenia, more so than positive symptoms.^3^ Existing treatments focus on reducing positive symptoms principally through the use of antipsychotic medications, but neither these medications nor other treatments have major beneficial effects on cognition. Indeed, it has been argued that antipsychotics, particularly at high doses, may exacerbate cognitive impairment.^4^ Interventions, such as cognitive remediation therapy, have been shown to improve cognitive deficits to a limited extent but are not routinely available for most patients with schizophrenia.^5^

The underlying causes of cognitive impairment in schizophrenia have been contested because first described by Kraepelin^6^ but include factors secondary to illness-related behaviors (eg, substance abuse and poor nutrition) and drugs used in treating the disorder, eg, high-dose antipsychotics,^7^ anticholinergics,^8^ and benzodiazepines.^9^ Nonetheless the demonstration in longitudinal population-based studies that cognitive impairment exists before schizophrenia onset^10^ suggests a contribution from factors that are correlated with increased liability to the disorder, including those that are etiological. Furthermore, evidence that cognitive performance is impaired in the relatives of those with schizophrenia, and is heritable in these families,^11^ indicates a genetic contribution to cognitive impairment in schizophrenia, consistent with the neurodevelopmental hypothesis of the disorder.

Genome-wide association studies (GWAS) have proven to be an effective means of identifying risk alleles for schizophrenia.^12,13^ They have also identified common alleles that influence population variation in measures of cognitive ability, including IQ, as well as other proxy measures such as educational attainment (EA). Furthermore, GWAS have provided evidence for shared genetic contributions to many of these traits (schizophrenia, bipolar disorder [BD], major depressive disorder [MDD], IQ, and EA).^14–20^ Common variant GWAS have previously been performed on cognition in schizophrenia cases at smaller sample sizes.^21,22^

The aggregated common variant genetic liability for disorders and traits can be estimated in individuals by a metric known as the polygenic risk score (PRS). The PRS for schizophrenia has been shown to be weakly associated with IQ and cognition in population samples^23–26^ and appears to be associated with severity of negative, but not positive symptoms in those with schizophrenia.^27^ IQ PRS has been shown to be significantly associated with schizophrenia diagnosis in a case/control sample.^23^

To date, few studies have examined the influence of PRS on cognition in those with schizophrenia, and those that have been performed have used a restricted range of PRSs, generally in small samples, and have found no convincing evidence for an association between schizophrenia PRS and cognition.^28–30^ Aiming to obtain insights into the origins of cognitive impairment in those with schizophrenia, we report analyses of what we believe is the largest schizophrenia sample to date for which both cognitive and genetic data are available. We derived *g*, the “general intelligence factor,” as a measure of general cognitive ability,^31^ because it has been used successfully in population-based genetic studies,^15^ it captures substantial variance in cognitive ability, particularly in schizophrenia,^32^ and can be derived from a diverse array of cognitive tests across different studies.^33,34^

We performed a GWAS of *g* within schizophrenia cases and systematically examined the relationship between *g* and PRSs for psychiatric disorders and cognitive traits in multiple schizophrenia case samples, using meta-analysis to combine the results. We had 2 primary hypotheses. First, under the hypothesis that variation in cognitive impairment in schizophrenia is essentially a consequence of liability to the disorder, with greater impairment indicating greater liability, we predicted that the measure of liability to schizophrenia (schizophrenia PRS) would be negatively associated with cognitive performance in those with the disorder (Hypothesis 1). Alternatively, under the hypothesis that variation in cognitive performance in schizophrenia is driven by similar factors that influence cognition in the general population, albeit that variance occurs around a mean point that is lower as a consequence of the disorder, we predicted that cognition-related PRS (for IQ and EA) would be associated with cognition in those with schizophrenia (Hypothesis 2). We also investigated whether polygenic liability to BD and MDD were associated with cognition, testing these as negative controls, because both are adult disorders that genetically overlap with schizophrenia but do not show genetic correlation with IQ.^19^

## Methods

We amalgamated genetic and cognitive data from those with schizophrenia and schizoaffective disorder from available datasets that were part of the Schizophrenia Working Group of the Psychiatric Genomics Consortium (PGC), as well as additional samples from the European Union Gene-Environment Interaction consortium (EUGEI) and from Ireland and Cardiff that have not yet been included in the published work of the PGC.

### PGC Samples

Of the 11 datasets in this study, 8 were part of the 2014 PGC schizophrenia study (table 1).^13^ Genetic data accessed from PGC servers with permission of the individual study principal investigators.

**Table 1.** Sample Size and Details of Datasets Included in Study

Dataset name	In PGC2 SZ study?	Country/countries of origin	Number of study participants	Gender (% female)	Median age	Age range
Bonn/Mannheim	Yes	Germany	436	42	36	17–70
PAGES	Yes	Germany	148	37	39	19–70
CATIE	Yes	United States	350	23	43	18–65
Hubin	Yes	Sweden	77	30	45	25–70
TOP	Yes	Norway	286	43	29	17–62
GROUP sample 1	Yes	The Netherlands	309	23	25	16–52
GROUP sample 2	Yes	The Netherlands	119	24	25	15–45
Ireland (PGC samples)	Yes	Ireland	346	28	42	17–69
Ireland (additional samples)	No	Ireland	159	35	43	19–67
EU-GEI Work Package 2	No	France, Italy, Spain, the Netherlands, United Kingdom	156	28	30	17–59
Cardiff cognition	No	United Kingdom	648	38	43	17–74

Note: PGC, psychiatric genomics consortium; PAGES, phenomics and genomics sample; CATIE, clinical antipsychotic trials for intervention effectiveness; TOP, Tematisk Omrade Psykoser, GROUP, genetic risk and outcome of psychosis; EU-GEI, European Union Gene-Environment Interaction. Number of study participants refers to those with genomic, phenotypic and covariate data.

### PGC Genotype Data

The PGC datasets included 2071 genotyped individuals of European ancestry, with research-verified diagnoses of schizophrenia or schizoaffective disorder for whom we also had sufficient cognitive data to calculate *g*, the general cognition factor. We used the quality control parameters reported by the PGC consortium,^13^ excluding individuals of non-European ancestry based on PCA. The datasets we analyzed had been imputed using the 1000 Genomes phase 3 reference panel with the programs SHAPEIT for haplotype phasing and IMPUTE2 for imputation. Full details of sample collection, genotyping, quality control, and imputation are available in the associated article.^13^ After imputation, variants with an INFO score >0.1, minor allele frequency (MAF) >0.5% and missingness <2% were retained for further analysis.

### EUGEI and Additional Irish Samples

A total of 156 samples with schizophrenia and schizoaffective disorder collected and genotyped as part of Work Package 2 of the EUGEI study were included in the analysis (the European network of national schizophrenia networks studying gene-environment interactions, see http://www.eu-gei.eu/).^35^ These samples were recruited as first episode psychosis cases with a schizophrenia or schizoaffective disorder diagnosis based on Operational Criteria ratings, following a research interview and case note review.^35^ An additional 159 cases collected from centers across Ireland were included in the analysis; all had a *Diagnostic and Statistical Manual of Mental Disorders* (Fourth Edition) (*DSM-IV*) diagnosis of schizophrenia/schizoaffective disorder. For details of genotyping, quality control, and imputation, see supplementary information.

### CardiffCOGS Samples

We included 648 samples from the CardiffCOGS study with *DSM-IV* schizophrenia and schizoaffective disorder diagnoses, based on a SCAN interview^36^ and clinical note review ratings.^37^ For details of genotyping, quality control, and imputation, see supplementary information.

### Neuropsychological Assessment

Participants in all studies underwent formal neuropsychological testing, administered by trained researchers. Protocols and results from each sample have been independently published^38–44^ and we provide full details of testing procedures and batteries in supplementary information.

### Calculation of g

The cognitive tests available differed for each study sample (supplementary table 1). For a dataset to be included, we required tests from a minimum of 2 cognitive domains, having assigned cognitive tests to domains based on the approach taken by MATRICS.^45,46^ We then calculated *g* independently for each dataset using at most 3 tests from a particular cognitive domain. Subjects were excluded if they did not have valid scores for at least 2 cognitive tests. Outlier test scores were also excluded (supplementary information).

*g* was calculated from the cognitive test scores using multidimensional scaling (MDS), as implemented in the R “stats” package. Unlike principal component analysis (PCA), MDS can retain subjects with missing data while being mathematically analogous to PCA when data are complete. *g* was calculated as the first dimension produced by MDS analysis.

For 5 datasets, values of *g* were calculated using both MDS and PCA in samples with no missing data, and the results examined for correlation (see supplementary data and supplementary table 2 for more details). For PCA, the first principal component was taken to represent *g*. PCA- and MDS-derived estimates of *g* were highly correlated (|r| > 0.95 in all datasets), endorsing our selection of MDS to derive *g*. A version of the primary analysis using values of *g* derived from PCA (thus excluding missing data) was also performed.

For the EUGEI sample, Wechsler Adult Intelligence Scale (WAIS) IQ estimates were available. Given their high correlation with *g*, and also because WAIS IQ had been standardized across the multiple countries present in the EUGEI dataset, we used these scaled IQ scores for the EUGEI samples. This methodology follows the approach taken in equivalent research in nonclinical populations.^23^

### Genome-Wide Association Analysis of *g* and Meta-analysis

Mixed linear model association was performed genome-wide in each dataset using the program Genome-wide Complex Trait Analysis,^47,48^ which calculates a genetic relationship matrix (GRM) for all samples that are then used to correct for sample relatedness and population stratification. To prevent overcorrection due to the inclusion of truly associated variants in the GRM, a leave-one-chromosome-out model was used where the GRM used for association testing for any variant on a given chromosome was derived after excluding all variants on that chromosome. The association results for the 11 datasets were combined using a standard error weighted, fixed effects meta-analysis in METAL.^49^

### PRS Construction

PRSs were constructed from GWAS of 5 disorders or traits as training sets (supplementary table 3); schizophrenia, major depression (MDD), BD, EA (measured in “years in education”), and IQ.^13,18,19,50,51^ The schizophrenia training set was based on the PGC2 meta-analysis but excluded the cognitively informative samples used in this study for analysis of PRS and *g*. Clumping was performed in imputed best-guess genotypes for each dataset using PLINK (maximum *r*^2^ = 0.2, window size = 500 kb, minimum MAF= 10%, minimum INFO score = 0.7), and variants within regions of long-range LD (including the MHC) excluded.^52^ PRS were then constructed from best-guess genotypes using PLINK at 10 *P* value thresholds (*P*_T_ = 1, .5, .3, .2, .1, .05, .01, 1 × 10^–4^, 1 × 10^–6^, 5 × 10^–8^). We used *P*_T_ = .05 for our primary analyses, except for MDD, where we used *P*_T_ = 0.5 (supplementary information).

### Regression of g on PRS and Meta-analysis

The relationships between *g* and PRS were analyzed in each schizophrenia dataset using linear regression in R, with age, sex, and population principal components as covariates (supplementary table 4). PRS and *g* were normalized to have a mean of 0 and a standard deviation of 1, and so resulting effect size estimates give the number of standard deviations change in *g* for 1 standard deviation change in PRS. Results for each PRS were meta-analyzed across all datasets with a fixed-effects model using the metagen function in the “meta” package in R. *I*^2^ values and random effects meta-analysis *P* values were also calculated to examine the extent of heterogeneity in our sample.

To ensure that the results were not biased by samples with a small number of available cognitive tests, or by the use of WAIS IQ in place of *g* in the EUGEI sample, we also performed sensitivity analyses, which excluded the EUGEI sample, and also individuals from 2 of the samples (Mannheim/Bonn and Ireland) for whom we had data for only 2 cognitive tests. Inclusion in the regression model of an age by sex interaction term and a nonlinear effect of the age covariate produced consistent results.

Power calculations for the PRS analyses are presented in supplementary information. For all training sets except BD, our power to detect true effects was estimated to be over 99% (supplementary table 3).

### Independent Population Samples

To examine whether the results for PRS predicting cognition in schizophrenia cases were comparable with results in a population-based sample, we tested the association between the IQ PRS (Savage et al^19^) and IQ in an independent dataset, the second wave of the Biobank sample (*n* = 91 468, *P*_T_ = .05, IQ measure: fluid intelligence score, UK Biobank field ID: 20016). We also tested the association between SZ PRS (Pardinas et al^12^) and IQ in the complete Biobank sample (*n* = 133 437, *P*_T_ = .05; supplementary information).

The analytic methods followed those of the main schizophrenia analysis and used population principal components, age at cognitive assessment, and sex as covariates (supplementary table 4). UK Biobank analyses were conducted under project number 13310.

## Results

Consistent with other similarly sized GWAS of complex traits, no variants reached a genome-wide level of significance for association with *g*. (Supplementary figure 1—Manhattan plot; supplementary figure 2—Q-Q plot (λ = 1.027); supplementary table 5—top hits; results available at https://walters.psycm.cf.ac.uk/).

**Fig. 1.**
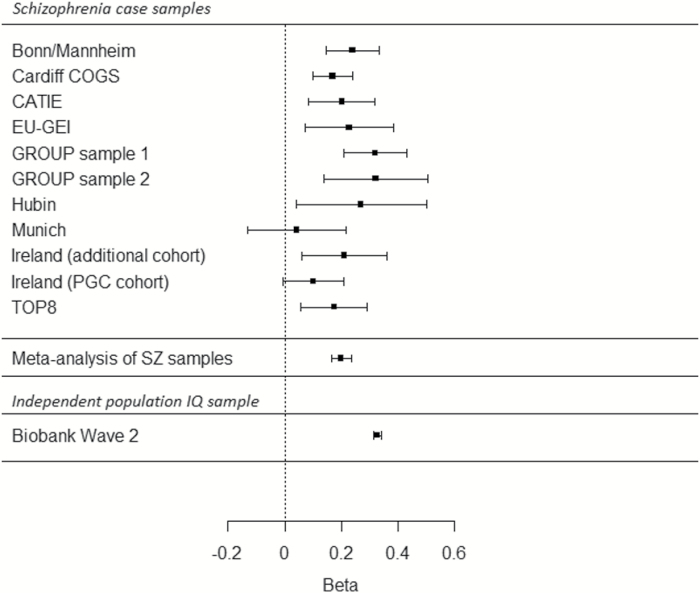
Forest plot showing effect sizes and confidence intervals for regression of *g* on IQ polygenic risk score (age, sex, and population principal component covariates also included in model) in schizophrenia case samples and an independent IQ sample. Effect sizes based on standardized values of *g*/IQ and polygenic risk score (PRS; ie, number of standard deviations change in *g*/IQ that occurs with 1 standard deviation change in PRS). Lower panel shows regression of IQ on IQ polygenic risk score in an independent population dataset, the second wave of the UK Biobank (*n* = 91 468).

With respect to our primary hypotheses, we found no evidence to support the predictions of hypothesis 1, in that we observed no association between the schizophrenia PRS and *g* in schizophrenia cases (table 2; supplementary figure 3). Thus, in our sample, common variant liability to schizophrenia was not associated with cognitive performance as measured by *g.* In contrast, a significant positive relationship was found between *g* and PRS derived from both IQ (*P* = 4.39 × 10^–28^, effect size = 0.199) and EA (*P* = 1.27 × 10^–26^, effect size = 0.188), supporting hypothesis 2 (table 2; figure 1; and supplementary figure 4). These effect sizes were larger in magnitude than those observed for SZ, BD, and MDD PRS, but somewhat smaller than those observed for the association of IQ PRS and fluid intelligence in non-psychotic individuals from the independent UK Biobank samples (*P* < 2.2 × 10^–16^, effect size = 0.327). Similar results were obtained across differing *P* value thresholds (supplementary table 6).

**Table 2.** Meta-analysis of Regression of *g* on PRS

Training set	*P* value threshold	Effect size	Standard error	*P* value
Schizophrenia	.05	–0.017	0.019	.386
Bipolar disorder	.05	–0.012	0.018	.509
Major depression	.5	–0.013	0.018	.488
IQ	.05	0.199	0.018	4.39E–28
Educational attainment	.05	0.188	0.018	1.27E–26

Sensitivity analysis following exclusion of the EUGEI samples (WAIS IQ was used instead of *g*) and samples with data on only 2 cognitive tests were consistent with the primary analyses (supplementary table 7). Similar results were observed when random effects meta-analysis was used to minimize the effect of intersample heterogeneity (supplementary table 6). The magnitude and pattern of results remained unchanged when the calculation of *g* used a traditional PCA approach (thus excluding participants with any missing cognitive test data). SZ PRS significantly predicted fluid intelligence in non-psychotic individuals in the Biobank sample (*P* < 2.2 × 10^–16^, effect size = –0.137), though again with a smaller effect size than when using IQ PRS.

Secondary negative control analyses revealed no significant relationship between *g* and PRS for BD or MDD (table 2; supplementary table 6).

## Discussion

Here, we report a genome-wide investigation of what is, to date, the largest schizophrenia sample with both cognitive and genetic data. Given that much larger samples are generally required to yield robust association signals for complex phenotypes and that this is true for general cognition in population samples,^23^ our aim was not to implicate loci associated with cognition within schizophrenia. Rather, our primary aim was to investigate the relationships between cognitive performance in people with schizophrenia and common variant genetic liability to both schizophrenia and to cognitive ability in the general population.

Specifically, we tested 2 primary hypotheses. First, under the hypothesis that variation in cognitive impairment in schizophrenia is a function of the degree of liability to the disorder, with greater impairment indicating greater liability, we predicted that the measure of liability to schizophrenia would be negatively associated with cognitive performance in those with the disorder. This hypothesis was not supported, as there was no significant relationship between schizophrenia PRS and *g*, although we cannot exclude the possibility that a significant relationship will emerge with further increases in sample size. The second hypothesis was that genetic variation in cognitive performance in schizophrenia is essentially driven by factors that influence cognition in the general population, leading to the prediction that cognition related PRS based on the general population would be associated with cognition in those with schizophrenia. In contrast to hypothesis 1, we found strong evidence to support the prediction from hypothesis 2, PRS for IQ and for EA being strongly associated with *g* in those with schizophrenia. As predicted, we found no evidence of association between liability to MDD or BPD and *g*.

Overall, our results suggest that alleles associated with IQ and EA in the general population make a more important contribution to variance in cognition in those with schizophrenia than the alleles that confer liability to schizophrenia per se. This interpretation, however, only holds if we assume the schizophrenia PRS captures a similar, or greater, proportion of the liability to that disorder than IQ and EA do for their respective traits. Previous studies have shown this assumption to be valid, indeed the IQ PRS explains a smaller proportion of variance in IQ than the proportion of variance of schizophrenia case status explained by the schizophrenia PRS (liability scale R^2^ = 0.052 for IQ, 0.07 for schizophrenia, 0.106–0.127 for EA).^13^ Thus, the schizophrenia PRS is actually better powered to test the impact of schizophrenia liability than the IQ PRS, allowing us to conclude that differential power is unlikely to explain our finding. Furthermore, the fact that the IQ and EA PRS predict cognition in cases indicates that the failure to detect a relationship between cognition and schizophrenia liability is not due to cognition measurement errors. Together, these considerations support the hypothesis that variance in cognition in schizophrenia and in the general population has common genetic causes.

We went on to examine whether the variance in cognition explained by the PRS for IQ was quantitatively as well as qualitatively similar in people with schizophrenia compared with those drawn from the wider population (figure 1). This showed that the IQ PRS explained less of the variance in cognition in schizophrenia than in an independent population sample (UK Biobank—UKBB^53^). We consider this to be only an approximate comparison of variance; an accurate comparison would require representative sampling at scale (population and case) and identical tests, neither condition being met in our schizophrenia sample. The IQ PRS was derived in large part from the UKBB (wave 1), which also provided our (nonoverlapping) independent test dataset for the population IQ analysis (wave 2 of UKBB). Thus, the observation that the variance explained in schizophrenia cases is modestly lower than in the UKBB population sample could be due, at least in part, to the more uniform cognitive assessment and similarity of sample characteristics (more restricted age range and demographics) in UKBB, which would serve to reduce unsystematic variation and increase power relative to the analysis in SZ cases. However, our result is also consistent with important contributions to cognitive impairment in those with schizophrenia from factors that are illness-related; possible examples include delays in treatment, symptom severity and chronicity, pre- and post-natal complications, social isolation, as well as drug exposures (therapeutic or abused).^7–9,54^

The fact that schizophrenia polygenic alleles *en masse* are not associated with variation in cognition in those with schizophrenia does not contradict previous findings that individual schizophrenia risk alleles or genes influence cognition or EA,^17^ indeed we and others have reported consistent negative associations between schizophrenia PRS and performance on specific cognitive domains and EA in population samples,^23,25^ and show here that schizophrenia PRS shows a negative association with cognition in the UKBB. The fact that we did not detect a similar negative association in cases may be partly attributable to the schizophrenia samples effectively having been selected for high schizophrenia PRS and thus attenuating our power to examine whether variation in schizophrenia PRS is associated with cognition. To examine this as a potential explanation of our results, we plotted the distributions and calculated metrics of normality for both the schizophrenia and IQ PRS (supplementary figures 5 and 6). These distributions and metrics are very similar between the schizophrenia and IQ PRS and are not suggestive of a restricted distribution, hence, although a theoretical concern, the distribution of schizophrenia PRS seems unlikely to explain our findings.

Our findings thus argue against universal pleiotropy for schizophrenia alleles and cognition. Nonetheless, our results do not suggest that schizophrenia risk alleles have no role in cognition that seems unlikely given the highly significant relationship between schizophrenia PRS and case/control status and the similarly robust cognitive impairments in cases relative to controls. Robust associations between SZ PRS and cognition in the general population, as we confirm, are further evidence against this. Rather, our findings suggest that the effect of schizophrenia risk alleles on cognition is well captured by the schizophrenia diagnosis. In other words, the schizophrenia PRS may contribute more to case-control cognitive differences than it does to the variance of cognition within cases, which is the subject of this study. The impact of schizophrenia alleles on cognitive functioning within cases must be small or absent and is certainly considerably less than the effect of alleles that contribute to IQ and EA PRS.

We acknowledge some limitations of our study design. Cross-sample cognitive analyses typically are hampered by differing test battery selection and administration. In this study, we sought to mitigate this by using *g* as a cognitive metric, which allows the incorporation of samples that use a diverse set of cognitive tests and has the benefit of ease of interpretation and comparison within and between studies. Despite this, heterogeneous effects related to test administration and sample ascertainment present challenges to combining cognitive data cross-site, although our findings suggest validity to our methods given the concordant results with equivalent population IQ studies. By conducting within sample PRS cognition analysis followed by meta-analysis, we also avoided the need to directly combine cognitive test results across samples. It is further reassuring that the subsets of our data do not show large amounts of variation in terms of the relationship between PRS and *g* (see forest plots in supplemental figures 3–4), and that cognitive PRS was in fact associated with *g* in our sample. Our study does not address the contribution of rare high-penetrance variants; however, although rare copy number variants and loss of function mutations clearly influence cognition and disorder liability, those that are currently known to do so are cumulatively so rare (2%–3% of cases) that they cannot contribute substantially to cognitive variance in the population of cases.^55,56^ Finally, we note our sample lacks matched healthy controls for whom similar cognitive data have been obtained, and therefore we cannot directly evaluate to what extent the cognitive PRS explains the average cognitive differences between those with and without the disorder. Despite the limitations of polygenic analysis with current sample sizes in explaining variance explained, it is unlikely that the major differences in cognition (1 to 2 standard deviations) seen between schizophrenia cases and healthy controls are explained by common genetic factors alone and that rare genetic variants and nongenetic exposures are likely to have important roles in etiology.

In conclusion, the existence of a genetic contribution to cognition in schizophrenia that is not secondary to the disorder per se has previously been inferred from findings that at least some of the cognitive impairment in people with schizophrenia predates the onset of the condition,^10^ and by the fact that cognitive impairments are observed, albeit in a milder form, in relatives of those with schizophrenia.^57^ We now extend these findings, showing for the first time that polygenic contribution to cognition overlaps in population and schizophrenia samples. We further show that in those with schizophrenia, variance in cognition is substantially independent of common variant liability to the disorder. This is important because it suggests the underlying biology of variation in cognition in schizophrenia will at least in part be elucidated through gaining insights into the genetic basis of cognition in population samples, and that such characterization may provide insights to inform the development of therapeutics for cognitive deficits in schizophrenia.

## Funding

Cardiff University researchers were supported by Medical Research Council (MRC) Centre (G0800509) and Programme Grant (G0801418). This study was supported by the NIMH PGC grant (5U01MH109514-02). The EU-GEI Project was funded by the European Community’s Seventh Framework Programme under grant agreement HEALTH-F2-2010–241909 (Project EU-GEI). This article represents independent research part funded by the National Institute for Health Research (NIHR) Biomedical Research Centre at South London and Maudsley National Health Service Foundation Trust and King’s College London.

## Supplementary Material

sbz061_suppl_Supplementary_Material

sbz061_suppl_Supplementary_Material_1
