# Supplementary material for: The Relationship Between Polygenic Risk Scores and Cognition in Schizophrenia
Source: Schizophr Bull. 2019 Jun 17;46(2):336–44. doi: 10.1093/schbul/sbz061 (PMC7442352; doi:10.1093/schbul/sbz061)
Supplement: sbz061_suppl_Supplementary_Material [file sbz061_suppl_supplementary_material.doc]

**Supplementary Information**

**Neuropsychological assessment**

Hubin Sample

Cognitive ability was assessed using the Cognitive Performance Indicator (CPI), a brief semi-computerized battery. Of CPI’s six domains, five were included in the following study using the following tasks:

1. Speed of processing (Trail Making Test: Part A and B)
2. Working memory (Letter-Number Span)
3. Attention / vigilance (Continuous Performance Test: Identical Pairs)
4. Verbal learning (Rey Auditory Verbal Learning Test (total))
5. Executive functioning (Wisconsin Card Sorting Test (64 card version):

Cases were recruited from northwestern Stockholm County and ascertainment has been described previously^1-6^. Cases gave informed consent and the human subjects protocol was approved by the ethical committees of the Karolinska Hospital and the Stockholm Regional Ethical Committee. Controls were recruited either among subjects previously participating in biological research at the Karolinska Institute or drawn from a representative register of the population of Stockholm County^2^. All participants provided informed consent.

TOP sample

Cognitive ability was assessed with a 3 h test battery. Tests from multiple domains of cognition were used to calculate *g*, including:

1. Speed of processing (WAIS Digit-Symbol Coding)^7^
2. Working memory (WAIS - Digit Span Test)^7^
3. Verbal memory (CVLT-II, WASI Vocabulary)^8, 9^
4. Visual memory (WASI Block Design)^9^
5. Executive function (WASI Matrix Reasoning ^9^

Participants were recruited as part of a large ongoing study on schizophrenia and bipolar disorder, the Thematic Organized Psychosis Research (TOP) Study, which is run from the University Hospitals in Oslo, Norway^10^. They were recruited from out-patient and in-patient psychiatric units at four University Hospitals in Oslo, Norway. The health care system is catchment area based, free of charge, and no other psychiatric health care provider exists. The patients were invited to participate in the study by the clinician responsible for their treatment. Healthy control participants were randomly selected from national statistical records from the same catchment area and contacted by letter inviting them to participate. Exclusion criteria for all groups were: IQ score below 70, hospitalized head injury, neurological disorder, unstable or uncontrolled medical condition that interferes with brain function (including hypothyroidism, uncontrolled hypertension and diabetes), outside the age range 17-65 years. To assure valid neurocognitive test performance all participants had to have Norwegian as their first language or have received their compulsory schooling in Norway, and had to score ≥15 on the forced recognition trial in the California Verbal Learning Test (CVLT-II)^8^. Neurocognitive assessment was carried out by psychologists trained in standardized neuropsychological testing. The test battery was administered in a fixed order with two breaks with refreshments.

Bonn-Mannheim Sample

Patients from Bonn and Mannheim were collected within the MooDS Consortium and were ascertained as previously described^11^. The study was carried out according to the ethical standards from Declaration of Helsinki. All participants gave written informed consent and the local ethics committees approved the study. Subjects were also part of the PGC schizophrenia data set^12^.

The applied instruments to assess cognitive ability differed between subsets of the sample. The present study used data from following domains and measures:

1. Speed of processing:

Trail Making Test - Part A^13^

1. Executive function:

Trail Making Test - Part B^13^

1. Working memory:

Letter-Number Span^14^

1. Verbal learning:

Verbal learning and memory test (VLMT)^15^

Scores from these tests were used to derive *g*.

GROUP cognitive dataset

Neuropsychological assessments were administered in a fixed order over a testing time of two hours^16-18^. These tests included:

1. Word learning task^18^
2. WAIS-III Digit Symbol Substitution test^17^
3. WAIS-III Information test^17^
4. WAIS-III Arithmetic test^17^
5. WAIS-III Block Design test^17^

These tests cover the majority of MATRICS domains, and the scores from them were used to derive *g*.

CATIE cognitive dataset

Cognitive ability was assessed using a selection of neurocognitive assessments chosen by an expert panel^19, 20^. Education level and the WRAT-III Reading subtest were administered. The following neurocognitive tests were administered.

1. Controlled Oral Word Association Test (Phonological Fluency)^21^

2. Semantic fluency (fruits, animals, vegetables categories)^21^

3. Wechsler Intelligence Scale for Children-third edition (WISC-m) Mazes^22^

4. Letter-Number Span Test^14^

5. Hopkins Verbal Learning Test^23^

6. WAIS-R Digit Symbol Test^24^

For each task, standardised z scores were derived by setting the mean of each measure to 0 and the standard deviation to 1 (across the whole patient sample)^20^. For selected measures, the performance of patients in this sample was compared to normative data derived from the general population. Scores from these six tests were then used to derive *g*.

Irish cognition dataset

Participants completed a full neuropsychological battery of measures to evaluate the cognitive deficits typically reported in schizophrenia (general cognitive function, episodic and working memory, attention, and social cognition). These tests included:

1. WAIS-III Vocabulary test^17^
2. WAIS-III Letter-Number sequencing test^17^
3. WMS-III Logical Memory test^17^

Scores from these tests were used to derive *g*.

Cardiff cognition dataset

Cognitive ability was assessed using the Measurement and Treatment Research to Improve Cognition in Schizophrenia (MATRICS) Consensus Cognitive Battery (MCCB)^25, 26^. Excluding the social cognition domain, the MCCB measures seven domains of cognition using nine tasks:

1. Speed of processing (Brief Assessment of Cognition in Schizophrenia: Symbol Coding; Semantic Fluency: Animals; Trail Making Test: Part A)

2. Working memory (Wechsler Memory Scale III: Spatial Span; Letter-Number Span)

3. Attention / vigilance (Continuous Performance Test: Identical Pairs)

4. Verbal learning (Hopkins Verbal Learning Test-Revised)

5. Visual learning (Brief Visuospatial Memory Test-Revised)

6. Executive function (Neuropsychological Assessment Battery: Mazes)

For each task, z scores were derived using the mean and standard deviation of the control group (50% males, mean age = 41.7 years). These scores were then used to derive the g measure of generalised cognition as described below.

PAGES — Phenomics and Genomics Sample cognitive dataset

Within PAGES (Phenomics and Genomics Sample; combined samples from Munich and Halle, Germany)^27^, unrelated outpatients or stable inpatients with a diagnosis of schizophrenia were ascertained from mental health services in the Munich. All participants were unrelated Caucasian middle Europeans. Detailed medical and psychiatric histories were collected, including the Structured Clinical Interview for DSM-IV (SCID), to evaluate lifetime Axis I and II diagnoses^28, 29^. Exclusion criteria included a history of head injury or neurological diseases. Participants were also rated for life time symptom severity using the PANSS^30^. Included patients were also part of the Psychiatric Genomics Consortium Schizophrenia Working Group dataset^12^.

Written informed consent was obtained from all participants. The study was approved by the local ethics committee of the Ludwig-Maximilians-University, Munich, Germany and carried out according to the ethical standards from Declaration of Helsinki.

All patients completed an extensive neuropsychological battery including the following tests:

1. Speed of processing:

Semantic (categories: food, animals) and phonemic verbal fluency (number of words starting with the letter S (P) in 60s), German version of the fluency test^31^

2. Attention and vigilance

3-7 Continuous Performance Test (d-prime, hits, false alarms)^32^

3. Verbal learning and memory

German version of the California verbal learning test (CVLT/VLMT) (immediate, delayed recall: raw score across 5 trials)^33^

4. Executive functioning

Tower of London (total time across all trials)^34^

EU-GEI sample

An abbreviated version of the WAIS, adapted from the most recent version available in each country was used in cases and controls in order to estimate IQ scores^35^. All versions included Digit Symbol substitution, Arithmetic, Block Design and Information subtests from which raw and scaled scores were derived. An estimated sum of full IQ scaled scores was calculated from the sum of available scaled scores (11/4*sum of scaled scores) and then converted to IQ, by using appropriate tables, standardized for each country. WAIS full scale IQ was derived from four subtests that are also able to estimate cognitive domains, described as follows^36^:

1. Block Design: Visuo-spatial learning and memory
2. Arithmetic: Working memory
3. Digit Symbol Substitution: Processing speed
4. Information: Verbal learning and memory

**Exclusion of outlier cognitive test scores**

All outlier test scores (those outside three standard deviations of the mean) were manually checked and excluded if they appeared invalid based on the ranges of possible scores on a particular test or if the score was inconsistent with scores on other tests (i.e. scores outside two standard deviations of the mean from other tests).

**Genotyping, genotype quality control and imputation of EUGEI and additional Irish samples**

These were genotyped at the MRC Centre for Neuropsychiatric Genetics and Genomics in Cardiff (UK) using a custom Illumina HumanCoreExome-24 BeadChip genotyping array covering 570,038 genetic variants^25^. SNP quality control exclusion parameters were: missingness >2%, Hardy Weinberg Equilibrium p<10^-6^. Samples with >2% missingness, heterozygosity F_het_ >0.14 or <-0.11, who failed gender checks or who clustered with non-European samples in PCA analysis were excluded, as were one member of each pair with a relatedness coefficient above 0.2. Genotypes were imputed on the Michigan Imputation Server using the Haplotype Reference Consortium reference panel (version 1.1) and the programs Eagle for haplotype phasing and Minimac3 for imputation^37-39^. After imputation, variants with an imputation r2 > 0.6, MAF > 0.1% and missingness < 1% were retained for further analysis.

**Genotyping, genotype quality control and imputation of CardiffCOGS samples**

The CardiffCOGS samples were genotyped on Illumina HumanOmniExpress-12 and OmniExpressExome-8 arrays and then quality controlled as previously described^40^. SNP quality control exclusion parameters were: missingness >2% and Hardy Weinberg Equilibrium p<10^-6^. Samples with >2% missingness, who failed gender checks or who appeared non-European in PCA analysis were excluded, as were one member of each pair with a relatedness coefficient above 0.2. The dataset was imputed using the 1000 Genomes phase 3 reference panel with the programs SHAPEIT for haplotype phasing and IMPUTE2 for imputation^41-43^. Variants with INFO >0.3 and MAF >0.1% were retained for further analysis.

**Choice of P_T_ threshold for primary analysis**

Schizophrenia PRS using P_T_=0.05 is the median and modal threshold that maximally explains variance in schizophrenia case/control status in the leave-one-out analysis in the PGC2 SZ study^12^. Similarly, bipolar disorder PRS using P_T_=0.05 explained the most variance in bipolar disorder case/control status in any leave-one-out analysis in the PGC BD study^44^. Hence, P_T_=0.05 was used for the primary analysis in SZ and BD. In the PGC major depressive disorder GWAS, P_T_=0.5 explained the most variance in all the replication analyses and so was used for the primary analysis^45^.

In the IQ study, IQ PRS using P_T_=0.058 explained the most variance in their largest replication analysis ^46^. The closest threshold we examined was P_T_=0.05, so we used this as our primary analysis. In the educational attainment study, only P_T_=1, 5x10^-8^, 5x10^-5^ and 5x10^-3^ were used in replication analyses, with the most variance being explained by P_T_=1 and P_T_=5x10^-3^ ^47^. As P_T_=0.05 fell between these two values and we wished to treat EA and IQ in a parallel manner given their phenotypic similarities, we used P_T_=0.05 for the primary analysis of EA.

**PRS power calculations**

The R package AVENGEME was used to estimate the expected power of the PRS of the training sets to predict cognition in a dataset this size^48, 49^, under the assumptions that cognitive variation in cases has similar heritability and cross-trait genetic correlations as IQ in the general population. These assumptions, which essentially correspond to power to test hypothesis 2, were required as little is known about the relevant parameters within cases. For all training sets except BD, our power to detect true effects was estimated to be over 99% (Supplementary Table 3). For BD, the lower genetic correlation with cognition meant our estimated power was 10.8%.

**Defining schizophrenia, bipolar disorder and psychotic disorder in UK Biobank Wave 2 sample**

Samples with a diagnosis of bipolar disorder, schizophrenia, or other psychosis were excluded from UK Biobank Wave 2 PRS analysis. We searched for evidence of a diagnosis of schizophrenia, bipolar affective disorder, and psychotic disorder from numerous sources within UK Biobank. Individuals were classed as having one of these disorders if there was any indication from any of the following sources (i) self-reported diagnosis at the assessment centre interview (UKBB field ID: 20002), (ii) an ICD-10 primary (UKBB field ID: 41202) or secondary (UKBB field ID: 41204) diagnosis from linked hospital records, (iii) an ICD-10 diagnosis from death records (UKBB field IDs: 40001 and 40002), or (iv) a self-reported diagnosis by a professional in the mental health questionnaire (MHQ) (UKBB field ID: 20544). The ICD-10 codes used for schizophrenia included F20 and F25, for bipolar affective disorder we included F30 and F31, and for psychotic disorder we included F21, F22, F23, F28 and F29.

**Constructing IQ PRS in UK Biobank Wave 2 sample**

The training set for the IQ PRS in Biobank was a version of Savage *et al* with Biobank samples excluded^46^. The training set for the SZ PRS was taken from Pardinas *et al^40^*. The PRS was calculated in PRSice (v2) using imputation dosage data for each UK Biobank participant that passed QC measures^50^. One member from each related pair with a kinship coefficient > 0.15 was excluded at random and analyses were restricted to individuals with European genetic ancestry. We selected high quality SNPs to calculate the PRS: INFO > 0.9, MAF > 0.1, missingness < 0.05, Hardy-Weinberg equilibrium (HWE) < 1 x 10^-6^, removed indels, and excluded the extended MHC region (25 MB – 35 MB). A reference panel of 1000 randomly selected UK Biobank participants was used to obtain relatively independent SNPs (r^2^ < 0.2, window size < 500kb).

**Supplementary Figures**

**
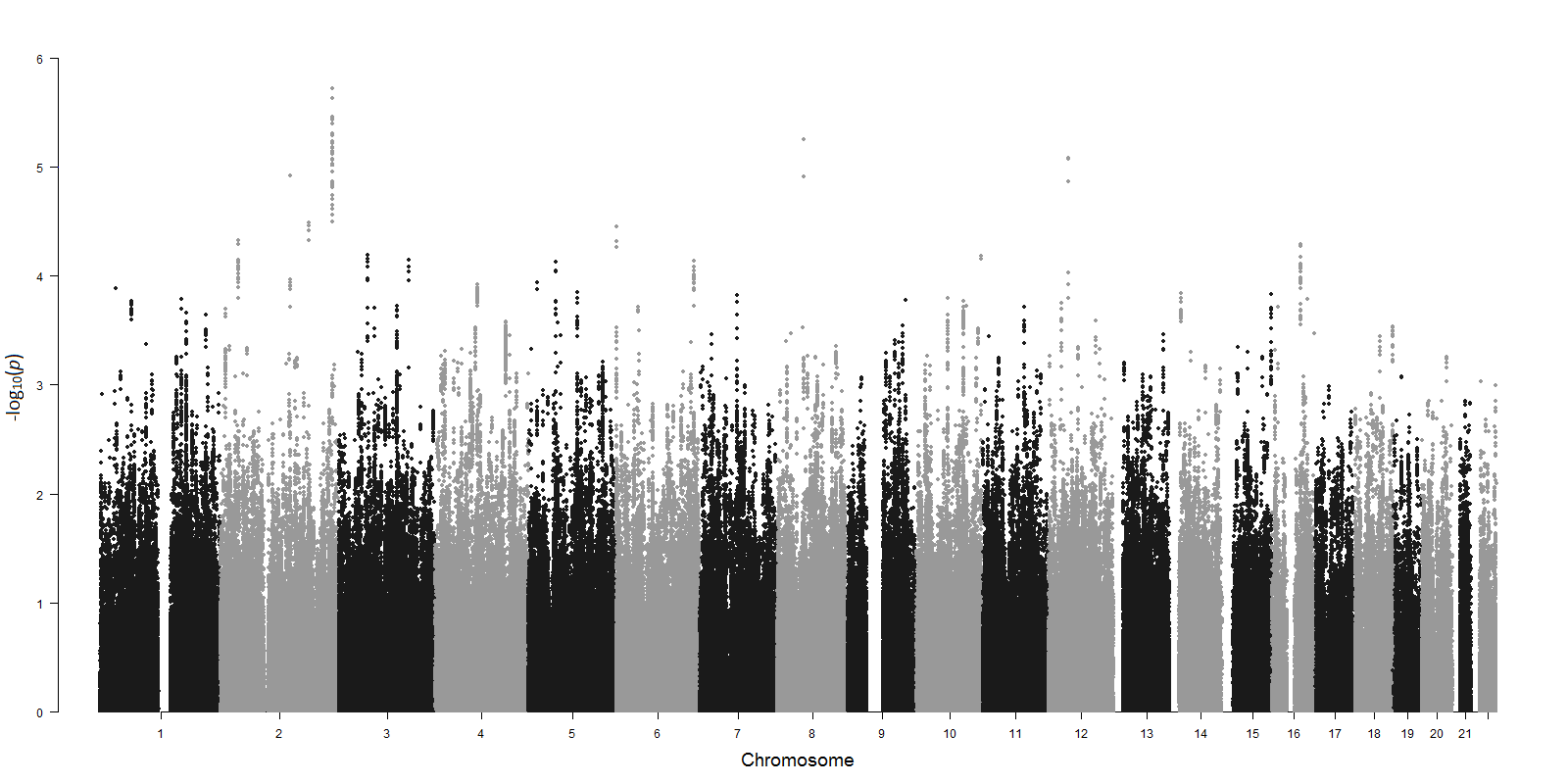
**

Supplementary Figure 1. Manhattan plot of GWAS of *g* meta-analysis


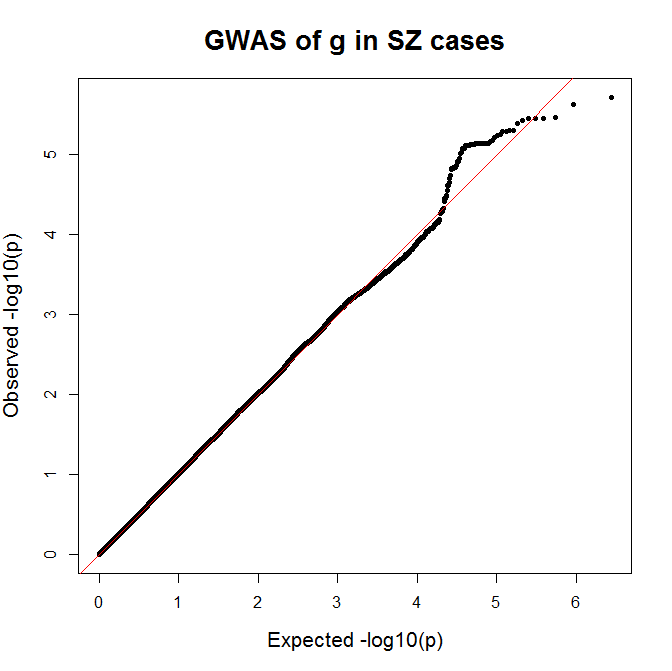


Supplementary Figure 2. Quantile-quantile plot of GWAS of *g* meta-analysis


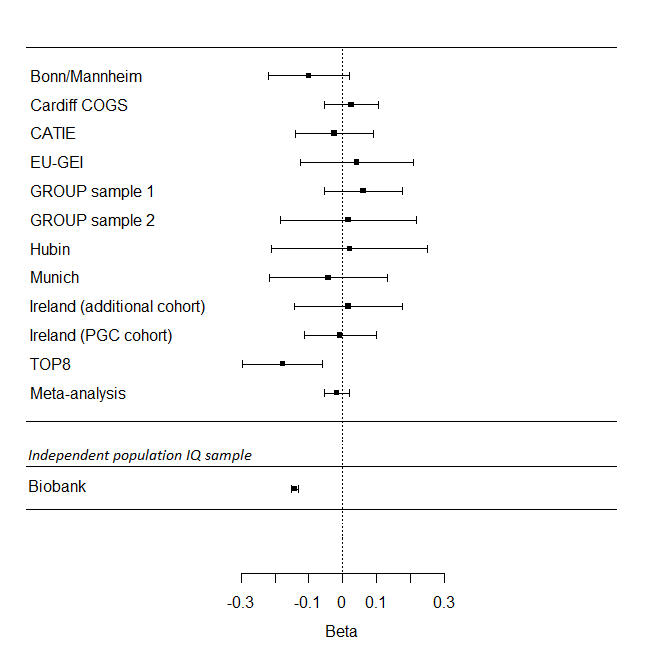


Supplementary Figure 3. Forest plot showing effect sizes and confidence intervals for regression of *g* on schizophrenia polygenic risk score (age, sex and population principal component covariates also included in model). Effect sizes based on standardised values of *g*/IQ and PRS (effect size is the number of standard deviations change in *g*/IQ that occurs when PRS changes by 1 standard deviation). WAIS IQ used instead of *g* for EU-GEI dataset. Lower panel shows regression of IQ on SZ polygenic risk score in an independent population dataset, the UK Biobank (n=133437).


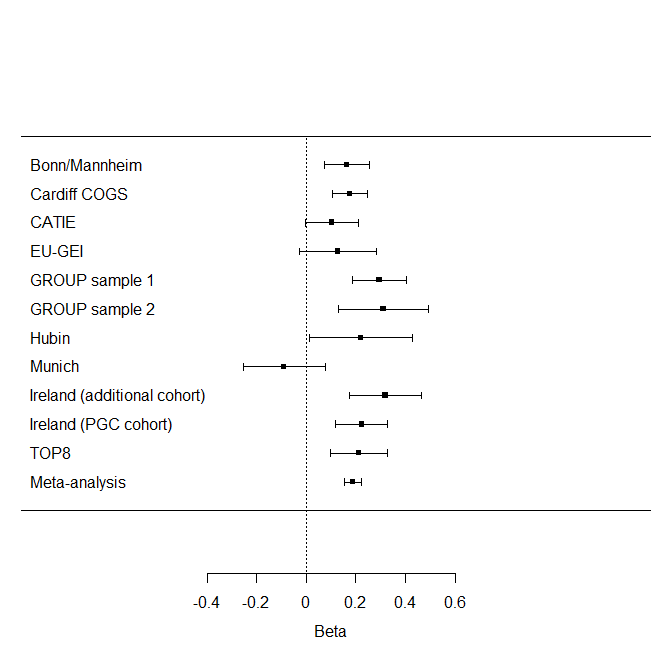


Supplementary Figure 4. Forest plot showing effect sizes and confidence intervals for regression of *g* on educational attainment polygenic risk score (age, sex and population principal component covariates also included in model). Effect sizes based on standardised values of *g*/IQ and PRS (effect size is the number of standard deviations change in *g*/IQ that occurs when PRS changes by 1 standard deviation). WAIS IQ used instead of *g* for EU-GEI dataset.

**
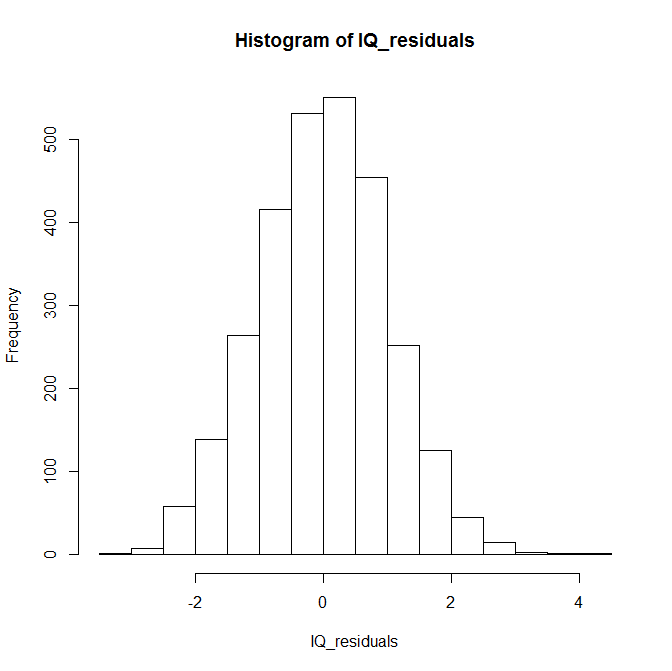
**

Supplementary Figure 5. Histogram of scaled IQ PRS residual values (after effects of covariates removed with linear regression) across all subsets of data. Shapiro-Wilks normality test p-value=0.053, kurtosis=3.02.

**
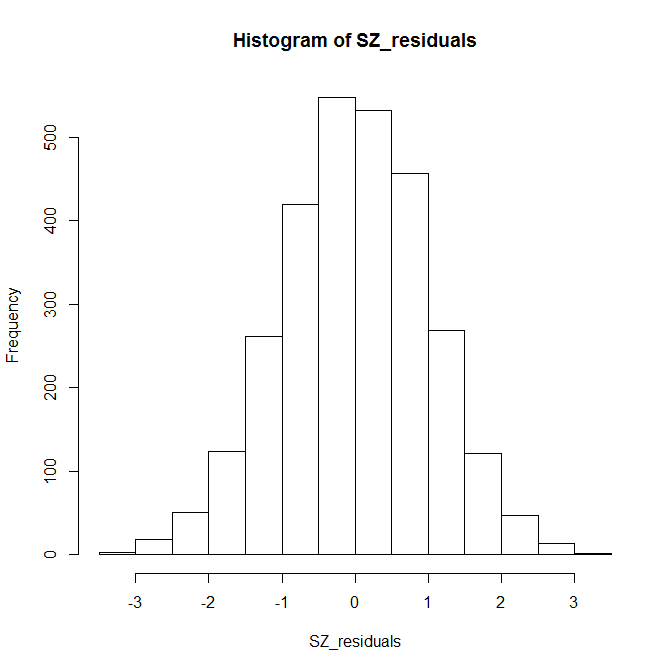
**

Supplementary Figure 6. Histogram of scaled SZ PRS residual values (after effects of covariates removed with linear regression) across all subsets of data. Shapiro-Wilks normality test p-value=0.567, kurtosis=2.91.

**Supplementary Tables**

| **Dataset** | **Cognitive domain** | **Test** | **N** |
| --- | --- | --- | --- |
| GROUP sample 1 | Speed of processing | WAIS digit symbol substitution | 309 |
|  | Verbal learning and memory | WAIS information | 309 |
|  |  | Brand and Jolles word learning task | 304 |
|  | Visuo-spatial learning and memory | WAIS block design | 308 |
|  | Working memory | WAIS arithmetic | 309 |
| GROUP sample 2 | Speed of processing | WAIS digit symbol substitution | 119 |
|  | Verbal learning and memory | WAIS information | 119 |
|  |  | Brand and Jolles word learning task | 117 |
|  | Visuo-spatial learning and memory | WAIS block design | 119 |
|  | Working memory | WAIS arithmetic | 119 |
| EU-GEI | Speed of processing | WAIS digit symbol substitution | 156 |
|  | Verbal learning and memory | WAIS information | 156 |
|  | Visuo-spatial learning and memory | WAIS block design | 156 |
|  | Working memory | WAIS arithmetic | 156 |
| CATIE | Executive function | WISC-m mazes | 342 |
|  | Speed of processing | WAIS digit symbol substitution | 348 |
|  |  | Semantic fluency (fruits, animals, vegetables categories) | 350 |
|  | Verbal learning and memory | Hopkins verbal learning test | 348 |
|  |  | Controlled oral word association test | 350 |
|  | Working memory | Letter number span | 347 |
| Cardiff cognition | Attention/Vigilance | Continuous Performance Test | 601 |
|  | Executive function | Mazes (NAB) | 647 |
|  | Speed of processing | Trail Making Test - part A | 647 |
|  |  | Semantic fluency (animals category) | 646 |
|  |  | Symbol Coding | 640 |
|  | Verbal learning and memory | Hopkins verbal learning test | 648 |
|  | Visuo-spatial learning and memory | Brief Visuospatial Memory Test Revised | 644 |
|  | Working memory | Letter number span | 642 |
|  |  | Spatial Span (WMS-III) | 645 |
|  | Attention/Vigilance | Continuous Performance Test | 77 |
|  | Executive function | Trail Making Test - part B | 77 |
|  |  | Wisconsin card sorting test | 77 |
|  | Speed of processing | Trail Making Test - part A | 77 |
|  | Verbal learning and memory | Rey Auditory Verbal Learning Test | 77 |
|  | Working memory | Letter number span | 77 |
| PAGES | Attention/Vigilance | Continuous Performance Test | 144 |
|  | Executive function | Tower of London | 145 |
|  | Speed of processing | Semantic fluency (food category) | 146 |
|  |  | Phonemic Fluency (words starting with letter 'X') | 146 |
|  | Verbal learning and memory | California Verbal Learning Test (immediate and delayed recall) | 148 |
| TOP | Executive function | WASI Matrix Reasoning | 285 |
|  | Speed of processing | WAIS Digit-Symbol Substitution | 285 |
|  | Verbal learning and memory | California Verbal Learning Test (delayed recall and total score) | 283 |
|  |  | WASI Vocabulary | 282 |
|  | Visuo-spatial learning and memory | WASI Block Design | 279 |
|  | Working memory | WAIS Digit Span | 286 |
| Ireland (PGC samples) | Verbal learning and memory | WAIS-III Vocabulary | 346 |
|  | Working memory | WMS-III Logical Memory | 333 |
|  |  | WAIS-III Letter number span | 326 |
| Ireland (additional samples) | Verbal learning and memory | WAIS-III Vocabulary | 162 |
|  | Working memory | WMS-III Logical Memory | 160 |
|  |  | WAIS-III Letter number span | 161 |
| Bonn/ Mannheim | Executive function | Trail Making Test - part B | 436 |
|  | Speed of processing | Trail Making Test - part A | 437 |
|  | Verbal learning and memory | Verbal learning and memory test (VLMT) | 196 |
|  | Working memory | Letter number span | 212 |

Supplementary Table 1. Cognitive domains and tests available in each dataset. N indicates number of genotyped samples available for each test.

| **Dataset** | **r** |
| --- | --- |
| Hubin | -0.99992 |
| PAGES | -0.99924 |
| TOP | 0.999965 |
| Ireland (PGC samples) | -0.99961 |
| Bonn/Mannheim | -0.95418 |

Supplementary Table 2. Correlation between values of *g* derived from MDS and PCA.

| **Phenotype** | **Training set** | **Sample size** | **Power** |
| --- | --- | --- | --- |
| Schizophrenia | PGC2 SZ (excluding cognitively informative samples)^12^ | 29958 cases, 39204 controls | 0.998 |
| Bipolar disorder | PGC Bipolar Disorder^44^ | 20352 cases, 31358 controls | 0.108 |
| Major depression | PGC MDD, Generation Scotland, GERA, deCODE, iPsych and UK Biobank meta-analysis^45^ | 51865 cases, 112200 controls | 1.000 |
| IQ | Savage *et al*^46^ | 269867 population samples | 1.000 |
| Educational attainment | Lee *et al*^47^ | 1131881 population samples | 1.000 |

Supplementary Table 3. Training sets for PRS construction. Power is calculated using the R package AVENGEME.

| **Dataset** | **Intelligence measure used** | **Predictor and covariates used** |
| --- | --- | --- |
| PGC datasets | *g* (derived by MDS) | Normalised PRS, age, sex, 10 population principal components |
| Cardiff COGS dataset | *g* (derived by MDS) | Normalised PRS, age, sex, 10 population principal components |
| Additional Irish samples | *g* (derived by MDS) | Normalised PRS, age, sex, 10 population principal components |
| EU-GEI WP2 dataset | WAIS IQ | Normalised PRS, age, sex, country, 5 population principal components |
| Biobank population dataset | Fluid intelligence | Normalised PRS, age, sex, 14 population principal components |

Supplementary Table 4. Parameters used in regression model. First 5 population principal components used for EU-GEI dataset. 10 population principal components used for PGC, COGS and additional Irish samples were those that showed association with SZ phenotype in PGC analysis^12^. 14 population principal components used for Biobank were those that showed association with fluid intelligence in Biobank.

| **SNP** | **CHR** | **BP** | **A1** | **A2** | **A1 Frequency** | **Effect size estimate** | **Standard error** | **P** |
| --- | --- | --- | --- | --- | --- | --- | --- | --- |
| rs911216 | 1 | 34375199 | A | C | 0.7963 | 0.1947 | 0.0509 | 0.0001317 |
| rs1556742 | 1 | 95674341 | A | C | 0.3493 | -0.1529 | 0.0433 | 0.0004215 |
| rs10919140 | 1 | 169296744 | A | T | 0.1446 | 0.2258 | 0.06 | 0.0001656 |
| rs16867210 | 2 | 10017166 | A | G | 0.241 | -0.1843 | 0.0495 | 0.0001999 |
| rs1396074 | 2 | 17339482 | T | C | 0.3233 | 0.1536 | 0.0437 | 0.0004424 |
| rs34439217 | 2 | 36656809 | A | T | 0.1477 | -0.2369 | 0.0582 | 4.72E-05 |
| rs17045898 | 2 | 54660795 | T | C | 0.1687 | 0.1977 | 0.0565 | 0.0004644 |
| rs2679441 | 2 | 142030276 | A | T | 0.2523 | -0.2096 | 0.0479 | 1.20E-05 |
| rs3106704 | 2 | 180486040 | T | C | 0.8929 | -0.2813 | 0.0677 | 3.29E-05 |
| rs60026510 | 2 | 228766207 | A | G | 0.5173 | 0.1974 | 0.0414 | 1.91E-06 |
| rs73837372 | 3 | 59536718 | T | G | 0.8086 | -0.2132 | 0.0534 | 6.49E-05 |
| rs12632137 | 3 | 72285695 | A | G | 0.6117 | 0.1597 | 0.0429 | 0.0001974 |
| rs6787458 | 3 | 119944395 | T | C | 0.0825 | -0.2848 | 0.0763 | 0.0001912 |
| rs6440154 | 3 | 142943025 | A | G | 0.3066 | -0.18 | 0.0453 | 7.14E-05 |
| rs114492241 | 4 | 19459837 | C | G | 0.0973 | -0.2455 | 0.0705 | 0.0004945 |
| rs2590820 | 4 | 54481514 | T | C | 0.5835 | -0.1452 | 0.0415 | 0.0004722 |
| rs116226341 | 4 | 82669798 | T | C | 0.1187 | 0.231 | 0.0639 | 0.0002986 |
| rs11097084 | 4 | 86880701 | T | C | 0.7702 | 0.1899 | 0.0494 | 0.0001192 |
| rs13134571 | 4 | 144659924 | T | G | 0.3277 | -0.1564 | 0.0429 | 0.0002644 |
| rs551305 | 4 | 152827837 | A | G | 0.5511 | -0.1477 | 0.0414 | 0.0003566 |
| rs181181726 | 5 | 6388840 | A | C | 0.0951 | 0.2479 | 0.0709 | 0.000474 |
| rs35181898 | 5 | 19081083 | T | C | 0.7911 | 0.194 | 0.0503 | 0.0001156 |
| rs96844 | 5 | 56196604 | A | G | 0.7454 | -0.1874 | 0.0473 | 7.52E-05 |
| rs7711530 | 5 | 61606610 | T | C | 0.6824 | -0.1635 | 0.0449 | 0.0002678 |
| rs190480 | 5 | 66133356 | T | C | 0.4811 | 0.1475 | 0.0413 | 0.0003498 |
| rs6596210 | 5 | 101287088 | T | C | 0.3918 | -0.163 | 0.0429 | 0.000142 |
| rs1161903 | 6 | 946027 | A | C | 0.1897 | 0.2169 | 0.0525 | 3.57E-05 |
| rs6902996 | 6 | 44475099 | A | T | 0.2499 | -0.1786 | 0.0479 | 0.0001955 |
| rs6914894 | 6 | 47695732 | T | C | 0.2162 | 0.1784 | 0.0496 | 0.0003196 |
| rs1781624 | 6 | 154126171 | A | G | 0.3083 | 0.1578 | 0.0446 | 0.0004083 |
| rs923198 | 6 | 159246717 | T | C | 0.4994 | -0.1638 | 0.0413 | 7.32E-05 |
| rs2938106 | 7 | 25580518 | T | C | 0.1123 | -0.2407 | 0.0673 | 0.0003463 |
| rs34236870 | 7 | 77547868 | T | C | 0.1164 | -0.2437 | 0.0643 | 0.0001519 |
| rs6997340 | 8 | 18286997 | T | C | 0.2768 | -0.1601 | 0.0452 | 0.0004032 |
| rs10095540 | 8 | 29355491 | T | G | 0.5466 | -0.15 | 0.0419 | 0.0003419 |
| rs7814396 | 8 | 55325204 | A | G | 0.9008 | 0.3207 | 0.0706 | 5.63E-06 |
| rs1714656 | 8 | 122006764 | A | G | 0.5931 | -0.1467 | 0.0417 | 0.0004406 |
| rs10992382 | 9 | 95361063 | A | C | 0.7796 | 0.1747 | 0.0493 | 0.0003891 |
| rs2786719 | 9 | 104598306 | C | G | 0.2675 | 0.1655 | 0.0468 | 0.0004048 |
| rs12683723 | 9 | 117913870 | A | G | 0.922 | 0.2961 | 0.0787 | 0.000167 |
| rs2136614 | 10 | 64701286 | A | G | 0.8601 | 0.2285 | 0.0606 | 0.0001615 |
| rs61886339 | 10 | 96032866 | T | C | 0.6963 | -0.1656 | 0.044 | 0.0001695 |
| rs7091572 | 10 | 101327851 | T | C | 0.7254 | 0.1718 | 0.046 | 0.0001905 |
| rs2459215 | 10 | 126089656 | T | G | 0.1825 | 0.194 | 0.0537 | 0.0003065 |
| rs7894208 | 10 | 132757095 | C | G | 0.1595 | -0.2297 | 0.0576 | 6.62E-05 |
| rs10500797 | 11 | 13724572 | T | C | 0.2809 | -0.1644 | 0.0461 | 0.0003618 |
| rs475639 | 11 | 85689785 | T | C | 0.5314 | -0.1545 | 0.0415 | 0.0001955 |
| rs79565578 | 12 | 26601174 | A | G | 0.9111 | 0.2772 | 0.074 | 0.0001783 |
| rs11177934 | 12 | 41099692 | A | G | 0.1357 | -0.2696 | 0.0605 | 8.35E-06 |
| rs10784131 | 12 | 61493136 | A | T | 0.612 | -0.1493 | 0.0426 | 0.0004562 |
| rs2032774 | 12 | 96651259 | T | C | 0.8863 | 0.2414 | 0.0661 | 0.0002593 |
| rs12306148 | 12 | 106060589 | T | G | 0.9027 | 0.2427 | 0.0694 | 0.0004687 |
| rs61966487 | 13 | 102662432 | T | C | 0.9274 | -0.2894 | 0.0809 | 0.0003482 |
| rs2331811 | 14 | 23199335 | A | G | 0.8564 | -0.226 | 0.0595 | 0.0001448 |
| rs11632769 | 15 | 33215074 | T | C | 0.3892 | 0.1484 | 0.0423 | 0.0004564 |
| rs624613 | 15 | 53716187 | A | G | 0.6534 | 0.1512 | 0.0434 | 0.0004972 |
| rs7180870 | 15 | 100621837 | A | T | 0.9405 | -0.3319 | 0.0875 | 0.0001482 |
| rs34864899 | 16 | 7986406 | A | G | 0.9054 | -0.2459 | 0.0704 | 0.0004781 |
| rs13339382 | 16 | 12269935 | A | G | 0.065 | 0.3168 | 0.085 | 0.0001935 |
| rs9930096 | 16 | 60284392 | A | G | 0.2356 | 0.1949 | 0.0481 | 5.13E-05 |
| rs12446726 | 16 | 74173688 | C | G | 0.1125 | 0.2496 | 0.0663 | 0.0001652 |
| rs58407170 | 16 | 87753841 | A | G | 0.8321 | -0.1953 | 0.0545 | 0.0003384 |
| rs6508210 | 18 | 50747191 | A | C | 0.4514 | 0.148 | 0.0415 | 0.0003597 |
| rs12961692 | 18 | 77508336 | T | C | 0.5154 | 0.1501 | 0.0415 | 0.0002946 |

Supplementary Table 5. Variants with association p<1e-4 in GWAS of *g.*

| **Training set** | **P-value threshold** | **Effect size (fixed effects)** | **SE (fixed effects)** | **P-value (fixed effects)** | **Meta-analysis I^2^** | **P-value (random effects)** |
| --- | --- | --- | --- | --- | --- | --- |
| Bipolar disorder | 1 | -0.015 | 0.019 | 0.426 | 43.8 | 0.528 |
| Bipolar disorder | 0.5 | -0.015 | 0.019 | 0.412 | 43.6 | 0.492 |
| Bipolar disorder | 0.3 | -0.012 | 0.019 | 0.510 | 30.5 | 0.632 |
| Bipolar disorder | 0.2 | -0.015 | 0.019 | 0.433 | 42.7 | 0.553 |
| Bipolar disorder | 0.1 | -0.025 | 0.019 | 0.177 | 45.3 | 0.425 |
| Bipolar disorder | 0.05 | -0.012 | 0.018 | 0.509 | 44.8 | 0.644 |
| Bipolar disorder | 0.01 | -0.006 | 0.018 | 0.732 | 34.8 | 0.848 |
| Bipolar disorder | 1.00E-04 | 0.001 | 0.018 | 0.976 | 0 | 0.976 |
| Bipolar disorder | 1.00E-06 | 0.005 | 0.018 | 0.767 | 0 | 0.767 |
| Bipolar disorder | 5.00E-08 | 0.015 | 0.018 | 0.387 | 0 | 0.387 |
| Major depression | 1 | -0.012 | 0.018 | 0.508 | 0 | 0.508 |
| Major depression | 0.5 | -0.013 | 0.018 | 0.488 | 0 | 0.488 |
| Major depression | 0.3 | -0.010 | 0.018 | 0.572 | 0 | 0.572 |
| Major depression | 0.2 | -0.001 | 0.018 | 0.947 | 0 | 0.947 |
| Major depression | 0.1 | 0.008 | 0.018 | 0.646 | 0 | 0.646 |
| Major depression | 0.05 | 0.009 | 0.018 | 0.598 | 0 | 0.598 |
| Major depression | 0.01 | 0.013 | 0.018 | 0.472 | 29.6 | 0.488 |
| Major depression | 1.00E-04 | -0.026 | 0.018 | 0.145 | 0 | 0.145 |
| Major depression | 1.00E-06 | 0.009 | 0.018 | 0.623 | 53.7 | 0.677 |
| Major depression | 5.00E-08 | 0.005 | 0.018 | 0.778 | 27 | 0.950 |
| Schizophrenia | 1 | -0.019 | 0.020 | 0.338 | 28.9 | 0.819 |
| Schizophrenia | 0.5 | -0.023 | 0.020 | 0.253 | 29.4 | 0.900 |
| Schizophrenia | 0.3 | -0.026 | 0.020 | 0.187 | 28.7 | 0.927 |
| Schizophrenia | 0.2 | -0.022 | 0.020 | 0.272 | 17.4 | 0.675 |
| Schizophrenia | 0.1 | -0.018 | 0.020 | 0.354 | 2.7 | 0.499 |
| Schizophrenia | 0.05 | -0.017 | 0.019 | 0.386 | 22.3 | 0.494 |
| Schizophrenia | 0.01 | -0.031 | 0.019 | 0.097 | 26 | 0.575 |
| Schizophrenia | 1.00E-04 | -0.026 | 0.018 | 0.149 | 29.1 | 0.660 |
| Schizophrenia | 1.00E-06 | -0.040 | 0.018 | 0.024 | 55.6 | 0.660 |
| Schizophrenia | 5.00E-08 | -0.019 | 0.018 | 0.275 | 34.3 | 0.853 |
| IQ | 1 | 0.198 | 0.018 | 3.36E-27 | 20.5 | 1.31E-20 |
| IQ | 0.5 | 0.198 | 0.018 | 2.81E-27 | 13.6 | 1.16E-22 |
| IQ | 0.3 | 0.202 | 0.018 | 2.45E-28 | 27.7 | 2.69E-19 |
| IQ | 0.2 | 0.202 | 0.018 | 2.17E-28 | 21.6 | 4.66E-21 |
| IQ | 0.1 | 0.201 | 0.018 | 1.64E-28 | 31.5 | 1.92E-18 |
| IQ | 0.05 | 0.199 | 0.018 | 4.39E-28 | 31.6 | 2.13E-18 |
| IQ | 0.01 | 0.177 | 0.018 | 4.20E-23 | 39 | 7.16E-14 |
| IQ | 1.00E-04 | 0.132 | 0.018 | 1.15E-13 | 44 | 4.46E-08 |
| IQ | 1.00E-06 | 0.098 | 0.018 | 4.04E-08 | 16.4 | 1.08E-06 |
| IQ | 5.00E-08 | 0.089 | 0.018 | 7.43E-07 | 0 | 7.43E-07 |
| Educational attainment | 1 | 0.183 | 0.018 | 3.28E-25 | 52.7 | 2.21E-11 |
| Educational attainment | 0.5 | 0.182 | 0.018 | 7.43E-25 | 54.5 | 8.07E-11 |
| Educational attainment | 0.3 | 0.185 | 0.018 | 7.13E-26 | 54.1 | 2.57E-11 |
| Educational attainment | 0.2 | 0.182 | 0.018 | 5.06E-25 | 54 | 4.81E-11 |
| Educational attainment | 0.1 | 0.187 | 0.018 | 2.34E-26 | 59.7 | 2.94E-10 |
| Educational attainment | 0.05 | 0.188 | 0.018 | 1.27E-26 | 57.9 | 5.02E-11 |
| Educational attainment | 0.01 | 0.178 | 0.018 | 5.37E-24 | 61.2 | 1.79E-09 |
| Educational attainment | 1.00E-04 | 0.15 | 0.018 | 2.22E-17 | 47.9 | 8.11E-09 |
| Educational attainment | 1.00E-06 | 0.145 | 0.018 | 2.43E-16 | 47.7 | 8.32E-08 |
| Educational attainment | 5.00E-08 | 0.132 | 0.018 | 1.18E-13 | 40.3 | 2.83E-07 |

Supplementary Table 6. Meta-analysis of regression of *g* on PRS across multiple p-value thresholds.

| **Training set** | **P-value threshold** | **Effect size** | **SE** | **P-value** |
| --- | --- | --- | --- | --- |
| Bipolar disorder | 0.05 | -0.012 | 0.02 | 0.54 |
| Major depression | 0.05 | 0.010 | 0.02 | 0.62 |
| Schizophrenia | 0.05 | -0.011 | 0.02 | 0.61 |
| IQ | 0.05 | 0.202 | 0.02 | 1.53E-25 |
| Educational attainment | 0.05 | 0.201 | 0.02 | 8.46E-27 |

Supplementary Table 7. Meta-analysis of regression of *g* on PRS at P_T_=0.05, sensitivity analysis excluding the EU-GEI dataset and samples with two or fewer cognitive tests available

References

**1.** Ekerholm M, Firus Waltersson S, Fagerberg T, Soderman E, Terenius L, Agartz I, Jonsson EG, Nyman H. Neurocognitive function in long-term treated schizophrenia: a five-year follow-up study. *Psychiatry research* Dec 30 2012;200(2-3):144-152.

**2.** Jonsson EG, Edman-Ahlbom B, Sillen A, et al. Brain-derived neurotrophic factor gene (BDNF) variants and schizophrenia: an association study. *Progress in neuro-psychopharmacology & biological psychiatry* Jul 2006;30(5):924-933.

**3.** Lawyer G, Nyman H, Agartz I, Arnborg S, Jonsson EG, Sedvall GC, Hall H. Morphological correlates to cognitive dysfunction in schizophrenia as studied with Bayesian regression. *Bmc Psychiatry* 2006;6.

**4.** Nesvag R, Frigessi A, Jonsson EG, Agartz I. Effects of alcohol consumption and antipsychotic medication on brain morphology in schizophrenia. *Schizophrenia research* Feb 2007;90(1-3):52-61.

**5.** Vares M, Ekholm A, Sedvall GC, Hall H, Jonsson EG. Characterization of patients with schizophrenia and related psychoses: Evaluation of different diagnostic procedures. *Psychopathology* 2006;39(6):286-295.

**6.** Ekholm B, Ekholm A, Adolfsson R, Vares M, Osby U, Sedvall GC, Jonsson EG. Evaluation of diagnostic procedures in Swedish patients with schizophrenia and related psychoses. *Nordic journal of psychiatry* 2005;59(6):457-464.

**7.** Wechsler D. *Wechsler Adult Intelligence Scale - Third Edition (WAIS-III). Norwegian manual.* Stockholm: Pearson Assessment; 2003.

**8.** Delis DC, Kramer JH, Kaplan E, Ober BA. *California Verbal Learning Test - Second Edition (CVLT-II). Norwegian Manual supplement.* Stockholm: Pearson Assessment; 2004.

**9.** Wechsler D. *Wechsler Abbreviated Scale of Intelligence (WASI). Norwegian manual supplement.* Stockholm: Pearson Assessment; 2007.

**10.** Athanasiu L, Mattingsdal M, Kahler AK, et al. Gene variants associated with schizophrenia in a Norwegian genome-wide study are replicated in a large European cohort. *Journal of psychiatric research* Sep 2010;44(12):748-753.

**11.** Stefansson H, Ophoff RA, Steinberg S, et al. Common variants conferring risk of schizophrenia. *Nature* Aug 6 2009;460(7256):744-747.

**12.** Schizophrenia Working Group of the Psychiatric Genomics C. Biological insights from 108 schizophrenia-associated genetic loci. *Nature* Jul 24 2014;511(7510):421-427.

**13.** Arnett JA, Labovitz SS. Effect of Physical Layout in Performance of the Trail Making Test. *Psychol Assessment* Jun 1995;7(2):220-221.

**14.** Gold JM, Carpenter C, Randolph C, Goldberg TE, Weinberger DR. Auditory working memory and Wisconsin Card Sorting Test performance in schizophrenia. *Archives of general psychiatry* Feb 1997;54(2):159-165.

**15.** Helmstaedter C, Durwen HF. [The Verbal Learning and Retention Test. A useful and differentiated tool in evaluating verbal memory performance]. *Schweizer Archiv fur Neurologie und Psychiatrie* 1990;141(1):21-30.

**16.** Korver N, Quee PJ, Boos HB, Simons CJ, de Haan L, investigators G. Genetic Risk and Outcome of Psychosis (GROUP), a multi-site longitudinal cohort study focused on gene-environment interaction: objectives, sample characteristics, recruitment and assessment methods. *International journal of methods in psychiatric research* Sep 2012;21(3):205-221.

**17.** Wechsler D. *WAIS‐III: Wechsler Adult Intelligence Scale (3rd edition) Administration and Scoring Manual*. San Antonio, TX: Psychological Corporation; 1997.

**18.** Brand N, Jolles J. Learning and retrieval rate of words presented auditorily and visually. *The Journal of general psychology* Apr 1985;112(2):201-210.

**19.** Keefe RS, Mohs RC, Bilder RM, Harvey PD, Green MF, Meltzer HY, Gold JM, Sano M. Neurocognitive assessment in the Clinical Antipsychotic Trials of Intervention Effectiveness (CATIE) project schizophrenia trial: development, methodology, and rationale. *Schizophrenia bulletin* 2003;29(1):45-55.

**20.** Keefe RS, Bilder RM, Harvey PD, et al. Baseline neurocognitive deficits in the CATIE schizophrenia trial. *Neuropsychopharmacology : official publication of the American College of Neuropsychopharmacology* Sep 2006;31(9):2033-2046.

**21.** Benton AL, Hamscher K. *Multilingual Aphasia Examination Manual (revised)*. Iowa City, LA: University of Iowa; 1978.

**22.** Wechsler D. *Wechsler Intelligence Scale for Children*. San Antonio, TX: Psychological Corporation; 1991.

**23.** Brandt J. The hopkins verbal learning test: Development of a new memory test with six equivalent forms. *Clinical Neuropsychologist* 1991;5(2):125-142.

**24.** Wechsler D. *Wechsler Adult Intelligence Scale-Revised*. San Antonio, TX: Psychological Corporation; 1974.

**25.** Nuechterlein KH, Green MF, Kern RS, et al. The MATRICS Consensus Cognitive Battery, part 1: test selection, reliability, and validity. *The American journal of psychiatry* Feb 2008;165(2):203-213.

**26.** Kern RS, Nuechterlein KH, Green MF, et al. The MATRICS Consensus Cognitive Battery, part 2: co-norming and standardization. *The American journal of psychiatry* Feb 2008;165(2):214-220.

**27.** Ingason A, Giegling I, Hartmann AM, et al. Expression analysis in a rat psychosis model identifies novel candidate genes validated in a large case-control sample of schizophrenia. *Translational psychiatry* Oct 13 2015;5:e656.

**28.** First M, Gibbon M, Spitzer R, Williams J, Benjamin L. *Structured Clinical Interview for DSM-IV Axis II Personality Disorders, (SCID-II)*. Washington, D.C.: American Psychiatric Press, Inc.; 1997.

**29.** First MB, Spitzer RL, Gibbon M, Williams JB. *Structured Clinical Interview for DSM-IV Axis I Disorders, Clinician Version (SCID-CV)*. Washington D.C.: American Psychiatric Press, Inc.; 1996.

**30.** Kay SR, Fiszbein A, Opler LA. The positive and negative syndrome scale (PANSS) for schizophrenia. *Schizophrenia bulletin* 1987;13(2):261-276.

**31.** Aschenbrenner S, Tucha O, Lange KW. *Regensburger verbal fluency test*: Hogsrefe; 2000.

**32.** Nuechterlein K, Arsanow R. *3-7 Continuous Performance Test*. Los Angeles: University of California; 2004.

**33.** Helmstaedter C, Lendt M, Lux S. *Verbaler Lern- und Merkfähigkeitstest (VLMT*. Goettingen: Beltz; 2001.

**34.** Tucha O, Lange KW. *TL- D Turm von London - Deutsche Version*. Goettingen: Hogrefe; 2004.

**35.** Ryan JJ, Lopez SJ, Werth TR. Administration time estimates for WAIS-III subtests, scales, and short forms in a clinical sample. *J Psychoeduc Assess* Dec 1998;16(4):315-323.

**36.** Taub GE, Benson N. Matters of Consequence: An Empirical Investigation of the WAIS-III and WAIS-IV and Implications for Addressing the Atkins Intelligence Criterion. *J Forensic Psychol P* Jan 1 2013;13(1):27-48.

**37.** Das S, Forer L, Schonherr S, et al. Next-generation genotype imputation service and methods. *Nature genetics* Oct 2016;48(10):1284-1287.

**38.** McCarthy S, Das S, Kretzschmar W, et al. A reference panel of 64,976 haplotypes for genotype imputation. *Nature genetics* Oct 2016;48(10):1279-1283.

**39.** Loh PR, Danecek P, Palamara PF, et al. Reference-based phasing using the Haplotype Reference Consortium panel. *Nature genetics* Nov 2016;48(11):1443-1448.

**40.** Pardinas AF, Holmans P, Pocklington AJ, et al. Common schizophrenia alleles are enriched in mutation-intolerant genes and in regions under strong background selection. *Nature genetics* Mar 2018;50(3):381-389.

**41.** Genomes Project C, Auton A, Brooks LD, et al. A global reference for human genetic variation. *Nature* Oct 1 2015;526(7571):68-74.

**42.** Delaneau O, Marchini J, Zagury JF. A linear complexity phasing method for thousands of genomes. *Nature methods* Dec 4 2011;9(2):179-181.

**43.** Howie BN, Donnelly P, Marchini J. A flexible and accurate genotype imputation method for the next generation of genome-wide association studies. *PLoS genetics* Jun 2009;5(6):e1000529.

**44.** Stahl EA, Breen G, Forstner AJ, et al. Genomewide association study identifies 30 loci associated with bipolar disorder. *bioRxiv* 2018(doi: 10.1101/173062 ).

**45.** Wray NR, Ripke S, Mattheisen M, et al. Genome-wide association analyses identify 44 risk variants and refine the genetic architecture of major depression. *Nature genetics* May 2018;50(5):668-681.

**46.** Savage JE, Jansen PR, Stringer S, et al. Genome-wide association meta-analysis in 269,867 individuals identifies new genetic and functional links to intelligence. *Nature genetics* Jul 2018;50(7):912-919.

**47.** Lee JJ, Wedow R, Okbay A, et al. Gene discovery and polygenic prediction from a genome-wide association study of educational attainment in 1.1 million individuals. *Nature genetics* Aug 2018;50(8):1112-1121.

**48.** Dudbridge F. Power and predictive accuracy of polygenic risk scores. *PLoS genetics* Mar 2013;9(3):e1003348.

**49.** Palla L, Dudbridge F. A Fast Method that Uses Polygenic Scores to Estimate the Variance Explained by Genome-wide Marker Panels and the Proportion of Variants Affecting a Trait. *American journal of human genetics* Aug 6 2015;97(2):250-259.

**50.** Euesden J, Lewis CM, O'Reilly PF. PRSice: Polygenic Risk Score software. *Bioinformatics* May 1 2015;31(9):1466-1468.
