# Supplementary material for: The Relationship Between Polygenic Risk Scores and Cognition in Schizophrenia
Source: Schizophr Bull. 2019 Jun 17;46(2):336–44. doi: 10.1093/schbul/sbz061 (PMC7442352; doi:10.1093/schbul/sbz061)
Supplement: sbz061_suppl_Supplementary_Material_1 [file sbz061_suppl_supplementary_material_1.docx]

**Genetic Risk and Outcome of Psychosis (GROUP) Investigators**

Behrooz Z. Alizadeh (a), Therese van Amelsvoort (g), Agna A. Bartels-Velthuis (a), Nico J. van Beveren (b, c, d), Richard Bruggeman (a), Wiepke Cahn (e), Lieuwe de Haan (f), Philippe Delespaul (g), Jurjen J. Luykx (e), Inez Myin-Germeys (h), Rene S. Kahn (e), Frederike Schirmbeck (f), Claudia J.P. Simons (g, i), Neeltje E. van Haren (e), Jim van Os (e, j), Ruud van Winkel (g,h)

a University of Groningen, University Medical Center Groningen, University Center for Psychiatry, Groningen, The Netherlands;

b Antes Center for Mental Health Care, Rotterdam, The Netherlands;

c Erasmus MC, Department of Psychiatry, Rotterdam, The Netherlands;

d Erasmus MC, Department of Neuroscience, Rotterdam, The Netherlands;

e University Medical Center Utrecht, Department of Psychiatry, Brain Centre Rudolf Magnus, Utrecht, The Netherlands;

f Academic Medical Center, University of Amsterdam, Department of Psychiatry, Amsterdam, The Netherlands;

g Maastricht University Medical Center, Department of Psychiatry and Psychology, School for Mental Health and Neuroscience, Maastricht, The Netherlands;

h KU Leuven, Department of Neuroscience, Research Group Psychiatry, Leuven, Belgium;

i GGzE Institute for Mental Health Care, Eindhoven, the Netherlands;

j King’s College London, King’s Health Partners, Department of Psychosis Studies, Institute of Psychiatry, London, United Kingdom

**The European Network of National Schizophrenia Networks Studying Gene-Environment Interactions (EU-GEI) WP2 Group**

Ulrich Reininghaus, PhD, Department of Psychiatry and Neuropsychology, School for Mental Health and Neuroscience, South Limburg Mental Health Research and Teaching Network, Maastricht University Medical Centre, Maastricht, the Netherlands, and Department of Health Service and Population Research, Institute of Psychiatry, King’s College London, De Crespigny Park, Denmark Hill, London, England; Marta Di Forti, MD, PhD, Department of Psychosis Studies, Institute of Psychiatry, King’s College London, De Crespigny Park, Denmark Hill, London, England; Kathryn Hubbard, MSc, Department of Health Service and Population Research, Institute of Psychiatry, King’s College London, De Crespigny Park, Denmark Hill, London, England; Stephanie Beards, PhD, Department of Health Service and Population Research, Institute of Psychiatry, King’s College London, De Crespigny Park, Denmark Hill, London, England; Simona A. Stilo, MSc, Department of Health Service and Population Research, and Department of Psychosis Studies, Institute of Psychiatry, King’s College London, De Crespigny Park, Denmark Hill, London, England; Giada Tripoli, MSc, Department of Psychosis Studies, Institute of Psychiatry, King’s College London, De Crespigny Park, Denmark Hill, London, England, and Department of Experimental Biomedicine and Clinical Neuroscience, Section of Psychiatry, University of Palermo, Palermo, Italy; Mara Parellada, MD, PhD, Department of Child and Adolescent Psychiatry, Hospital General Universitario Gregorio Marañón, School of Medicine, Universidad Complutense, Investigación Sanitaria del Hospital Gregorio Marañón (Centro de Investigación Biomédica en Red de Salud Mental), Madrid, Spain; Pedro Cuadrado, MD, Villa de Vallecas Mental Health Department, Villa de Vallecas Mental Health Centre, Hospital Universitario Infanta Leonor/Hospital Virgen de la Torre, Madrid, Spain; José Juan Rodríguez Solano, MD, Puente de Vallecas Mental Health Department, Hospital Universitario Infanta Leonor/Hospital Virgen de la Torre, Centro de Salud Mental Puente de Vallecas, Madrid, Spain; Angel Carracedo, MD, PhD, Fundación Pública Galega de Medicina Xenómica, Hospital Clínico Universitario, Santiago de Compostela, Spain; Enrique García Bernardo, MD, Department of Psychiatry, Hospital General Universitario Gregorio Marañón, School of Medicine, Universidad Complutense, Investigación Sanitaria del Hospital Gregorio Marañón (Centro de Investigación Biomédica en Red de Salud Mental), Madrid, Spain; Laura Roldán, PhD, Department of Child and Adolescent Psychiatry, Hospital General Universitario Gregorio Marañón, School of Medicine, Universidad Complutense, Investigación Sanitaria del Hospital Gregorio Marañón (Centro de Investigación Biomédica en Red de Salud Mental), Madrid, Spain; Gonzalo López, PhD, Department of Child and Adolescent Psychiatry, Hospital General Universitario Gregorio Marañón, School of Medicine, Universidad Complutense, Investigación Sanitaria del Hospital Gregorio Marañón (Centro de Investigación Biomédica en Red de Salud Mental), Madrid, Spain; Bibiana Cabrera, MD, Department of Psychiatry, Hospital Clinic, Institut d’Investigacions Biomèdiques August Pi i Sunyer, Centro de Investigación Biomédica en Red de Salud Mental, Universidad de Barcelona, Barcelona, Spain; Esther Lorente-Rovira, PhD, Department of Psychiatry, School of Medicine, Universidad de Valencia, Centro de Investigación Biomédica en Red de Salud Mental, Valencia, Spain; Paz Garcia-Portilla, MD, PhD, Department of Medicine, Psychiatry Area, School of Medicine, Universidad de Oviedo, Centro de Investigación Biomédica en Red de Salud Mental, Oviedo, Spain; Javier Costas, PhD, Fundación Pública Galega de Medicina Xenómica, Hospital Clínico Universitario, Santiago de Compostela, Spain; Estela Jiménez-López, MSc, Department of Psychiatry, Servicio de Psiquiatría Hospital “Virgen de la Luz,” Cuenca, Spain; Mario Matteis, MD, Department of Child and Adolescent Psychiatry, Hospital General Universitario Gregorio Marañón, School of Medicine, Universidad Complutense, Investigación Sanitaria del Hospital Gregorio Marañón (Centro de Investigación Biomédica en Red de Salud Mental), Madrid, Spain; Marta Rapado, PhD, Department of Child and Adolescent Psychiatry, Hospital General Universitario Gregorio Marañón, School of Medicine, Universidad Complutense, Investigación Sanitaria del Hospital Gregorio Marañón (Centro de Investigación Biomédica en Red de Salud Mental), Madrid, Spain; Emiliano González, PhD, Department of Child and Adolescent Psychiatry, Hospital General Universitario Gregorio Marañón, School of Medicine, Universidad Complutense, Investigación Sanitaria del Hospital Gregorio Marañón (Centro de Investigación Biomédica en Red de Salud Mental), Madrid, Spain; Covadonga Martínez, MD, Department of Child and Adolescent Psychiatry, Hospital General Universitario Gregorio Marañón, School of Medicine, Universidad Complutense, Investigación Sanitaria del Hospital Gregorio Marañón (Centro de Investigación Biomédica en Red de Salud Mental), Madrid, Spain; Emilio Sánchez, MD, Department of Psychiatry, Hospital General Universitario Gregorio Marañón, School of Medicine, Universidad Complutense, Investigación Sanitaria del Hospital Gregorio Marañón (Centro de Investigación Biomédica en Red de Salud Mental), Madrid, Spain; Mª Soledad Olmeda, MD, Department of Psychiatry, Hospital General Universitario Gregorio Marañón, School of Medicine, Universidad Complutense, Investigación Sanitaria del Hospital Gregorio Marañón (Centro de Investigación Biomédica en Red de Salud Mental), Madrid, Spain; Nathalie Franke, MSc, Department of Psychiatry, Early Psychosis Section, Academic Medical Centre, University of Amsterdam, Amsterdam, the Netherlands; Eva Velthorst, PhD, Department of Psychiatry, Early Psychosis Section, Academic Medical Centre, University of Amsterdam, Amsterdam, the Netherlands, and Departments of Psychiatry and Preventive Medicine, Icahn School of Medicine at Mount Sinai, New York, New York; Fabian Termorshuizen, PhD, Department of Psychiatry and Neuropsychology, School for Mental Health and Neuroscience, South Limburg Mental Health Research and Teaching Network, Maastricht University Medical Centre, Maastricht, the Netherlands, and Rivierduinen Centre for Mental Health, Leiden, the Netherlands; Daniella van Dam, PhD, Department of Psychiatry, Early Psychosis Section, Academic Medical Centre, University of Amsterdam, Amsterdam, the Netherlands; Elsje van der Ven, PhD, Department of Psychiatry and Neuropsychology, School for Mental Health and Neuroscience, South Limburg Mental Health Research and Teaching Network, Maastricht University Medical Centre, Maastricht, the Netherlands, and Rivierduinen Centre for Mental Health, Leiden, the Netherlands; Elles Messchaart, MSc, Rivierduinen Centre for Mental Health, Leiden, the Netherlands; Marion Leboyer, MD, PhD, AP-HP, Groupe Hospitalier “Mondor,” Pôle de Psychiatrie, Créteil, France, Institut National de la Santé et de la Recherche Médicale, U955, Créteil, France, Faculté de Médecine, Université Paris-Est, Créteil, France, and Fondation Fondamental, Créteil, France; Franck Schürhoff, MD, PhD, AP-HP, Groupe Hospitalier “Mondor,” Pôle de Psychiatrie, Créteil, France, Institut National de la Santé et de la Recherche Médicale, U955, Créteil, France, Faculté de Médecine, Université Paris-Est, Créteil, France, and Fondation Fondamental, Créteil, France; Stéphane Jamain, PhD, Institut National de la Santé et de la Recherche Médicale, U955, Créteil, France, Faculté de Médecine, Université Paris-Est, Créteil, France, and Fondation Fondamental, Créteil, France; Flora Frijda, MSc, Etablissement Public de Santé Maison Blanche, Paris, France; Grégoire Baudin, MSc, AP-HP, Groupe Hospitalier “Mondor,” Pôle de Psychiatrie, Créteil, France, Institut National de la Santé et de la Recherche Médicale, U955, Créteil, France; Aziz Ferchiou, MD, AP-HP, Groupe Hospitalier “Mondor,” Pôle de Psychiatrie, Créteil, France, Institut National de la Santé et de la Recherche Médicale, U955, Créteil, France; Baptiste Pignon, MD, AP-HP, Groupe Hospitalier “Mondor,” Pôle de Psychiatrie, Créteil, France, Institut National de la Santé et de la Recherche Médicale, U955, Créteil, France, and Fondation Fondamental, Créteil, France; Jean-Romain Richard, MSc, Institut National de la Santé et de la Recherche Médicale, U955, Créteil, France, and Fondation Fondamental, Créteil, France; Thomas Charpeaud, MD, Fondation Fondamental, Créteil, France, CMP B CHU, Clermont Ferrand, France, and Université Clermont Auvergne, Clermont-Ferrand, France; Anne-Marie Tronche, MD, Fondation Fondamental, Créteil, France, CMP B CHU, Clermont Ferrand, France, and Université Clermont Auvergne, Clermont-Ferrand, France; Daniele La Barbera, MD, PhD, Department of Experimental Biomedicine and Clinical Neuroscience, Section of Psychiatry, University of Palermo, Palermo, Italy; Caterina La Cascia, PhD, Department of Experimental Biomedicine and Clinical Neuroscience, Section of Psychiatry, University of Palermo, Palermo, Italy; Giovanna Marrazzo, MD, PhD, Unit of Psychiatry, “P. Giaccone” General Hospital, Palermo, Italy; Lucia Sideli, PhD, Department of Experimental Biomedicine and Clinical Neuroscience, Section of Psychiatry, University of Palermo, Palermo, Italy; Crocettarachele Sartorio, PhD, Unit of Psychiatry, “P. Giaccone” General Hospital, Palermo, Italy; Laura Ferraro, PhD, Department of Experimental Biomedicine and Clinical Neuroscience, Section of Psychiatry, University of Palermo, Palermo, Italy; Fabio Seminerio, MSc, Department of Experimental Biomedicine and Clinical Neuroscience, Section of Psychiatry, University of Palermo, Palermo, Italy; Camila Marcelino Loureiro, MD, Departamento de Neurociências e Ciencias do Comportamento, Faculdade de Medicina de Ribeirão Preto, Universidade de São Paulo, São Paulo, Brasil, and Núcleo de Pesquina em Saúde Mental Populacional, Universidade de São Paulo, São Paulo, Brasil; Rosana Shuhama, PhD, Departamento de Neurociências e Ciencias do Comportamento, Faculdade de Medicina de Ribeirão Preto, Universidade de São Paulo, São Paulo, Brasil, and Núcleo de Pesquina em Saúde Mental Populacional, Universidade de São Paulo, São Paulo, Brasil; Mirella Ruggeri, MD, PhD, Section of Psychiatry, Department of Neuroscience, Biomedicine and Movement, University of Verona, Verona, Italy; Sarah Tosato, MD, PhD, Section of Psychiatry, Department of Neuroscience, Biomedicine and Movement, University of Verona, Verona, Italy; Chiara Bonetto, PhD, Section of Psychiatry, Department of Neuroscience, Biomedicine and Movement, University of Verona, Verona, Italy; Doriana Cristofalo, MA, Section of Psychiatry, Department of Neuroscience, Biomedicine and Movement, University of Verona, Verona, Italy; Eva Velthorst, Department of Psychiatry, Icahn School of Medicine at Mount Sinai, New York, New York and Department of Psychiatry, Academic Medical Centre University of Amsterdam, Amsterdam, the Netherlands; Sheri Oduola, Department of Health Service and Population Research, King's College London, Institute of Psychiatry, Psychology & Neuroscience, London, UK and South London and Maudsley NHS Foundation Trust, NIHR Maudsley Biomedical Research Centre, London, UK.

**Schizophrenia Working Group of the Psychiatric Genomics Consortium**

Rolf Adolfsson, Ingrid Agartz, Esben Agerbo, Margot Albus, Madeline Alexander, Farooq Amin, Ole A Andreassen, Silviu A Bacanu, Martin Begemann, Richard A Belliveau Jr, Judit Bene, Sarah E Bergen, Elizabeth Bevilacqua, Tim B Bigdeli, Donald W Black, Douglas HR Blackwood, Anders D Børglum, Elvira Bramon, Richard Bruggeman, Nancy G Buccola, Randy L Buckner, Brendan Bulik-Sullivan, Joseph D Buxbaum, William Byerley, Wiepke Cahn, Guiqing Cai, Murray J Cairns, Dominique Campion, Rita M Cantor, Vaughan J Carr, Noa Carrera, Stanley V Catts, Kimberley D Chambert, Raymond CK Chan, Eric YH Chen, Ronald YL Chen, Wei Cheng, Eric FC Cheung, Siow Ann Chong, Sven Cichon, C Robert Cloninger, David Cohen, Nadine Cohen, David A Collier, Paul Cormican, Aiden Corvin, Nick Craddock, Benedicto Crespo-Facorro, James J Crowley, David Curtis, Mark J Daly, Ariel Darvasi, Michael Davidson, Kenneth L Davis, Franziska Degenhardt, Jurgen Del Favero, Lynn E DeLisi, Ditte Demontis, Dimitris Dikeos, Timothy Dinan, Srdjan Djurovic, Enrico Domenici, Gary Donohoe , Elodie Drapeau, Jubao Duan, Frank Dudbridge, Hannelore Ehrenreich, Peter Eichhammer, Johan Eriksson, Valentina Escott-Price, Tõnu Esko, Laurent Essioux, Ayman H Fanous, Kai-How Farh, Martilias S Farrell, Josef Frank, Lude Franke, Robert Freedman, Nelson B Freimer, Joseph I Friedman, Menachem Fromer, Pablo V Gejman, Giulio Genovese, Elliot S Gershon, Ina Giegling, Michael Gill , Paola Giusti-Rodríguez, Stephanie Godard, Jacqueline I Goldstein, Srihari Gopal, Jacob Gratten, Lieuwe de Haan, Christian Hammer, Marian L Hamshere, Mark Hansen, Thomas Hansen, Vahram Haroutunian, Annette M Hartmann, Frans A Henskens, Stefan Herms, Joel N Hirschhorn, Per Hoffmann, Andrea Hofman, Mads V Hollegaard, Peter A Holmans, David M Hougaard, Hailiang Huang, Christina M Hultman, Masashi Ikeda, Nakao Iwata, Assen V Jablensky, Inge Joa , Erik G Jönsson, Antonio Julià, Anna K Kähler, René S Kahn, Luba Kalaydjieva, Sena Karachanak-Yankova, Juha Karjalainen, David Kavanagh, Matthew C Keller, Brian J Kelly, Kenneth S Kendler, James L Kennedy, Andrey Khrunin, Yunjung Kim, George Kirov, Janis Klovins, Jo Knight, James A Knowles, Bettina Konte, Vaidutis Kucinskas, Zita Ausrele Kucinskiene, Hana Kuzelova-Ptackova, Max Lam, Claudine Laurent, Phil Lee, S Hong Lee, Jimmy Lee Chee Keong, Sophie E Legge, Todd Lencz, Bernard Lerer, Douglas F Levinson, Miaoxin Li, Tao Li, Qingqin S Li, Kung-Yee Liang, Jeffrey Lieberman, Svetlana Limborska, Jianjun Liu, Jouko Lönnqvist, Carmel M Loughland, Jan Lubinski, Milan Macek Jr, Patrik KE Magnusson, Brion S Maher, Wolfgang Maier, Anil K Malhotra, Jacques Mallet, Sara Marsal, Manuel Mattheisen, Morten Mattingsdal, Robert W McCarley, Steven A McCarroll, Colm McDonald, Andrew M McIntosh, Andrew McQuillin, Sandra Meier, Carin J Meijer, Bela Melegh, Ingrid Melle, Raquelle I Mesholam-Gately, Andres Metspalu, Patricia T Michie, Lili Milani, Vihra Milanova, Younes Mokrab, Jennifer L Moran, Derek W Morris, Ole Mors, Preben B Mortensen, Bryan J Mowry, Bertram Müller-Myhsok, Kieran C Murphy, Robin M Murray, Inez Myin-Germeys, Benjamin M Neale, Mari Nelis, Igor Nenadic, Deborah A Nertney, Gerald Nestadt, Kristin K Nicodemus, Liene Nikitina-Zake, Laura Nisenbaum, Annelie Nordin, Nina Norgren, Markus M Nöthen, Eadbhard O'Callaghan, Michael C O'Donovan, Colm O'Dushlaine, F Anthony O'Neill, Sang-Yun Oh, Ann Olincy, Line Olsen, Roel A Ophoff, Jim Van Os, Michael J Owen, Sara A Paciga, Aarno Palotie, Christos Pantelis, George N Papadimitriou, Sergi Papiol, Elena Parkhomenko, Michele T Pato, Carlos N Pato, Tiina Paunio, Diana O Perkins, Tune H Pers, Tracey L Petryshen, Olli Pietiläinen, Jonathan Pimm, Andrew J Pocklington, Danielle Posthuma, John Powell, Alkes Price, Ann E Pulver, Shaun M Purcell, Digby Quested, Henrik B Rasmussen, Abraham Reichenberg, Mark A Reimers, Alexander L Richards, Marcella Rietschel, Brien P Riley, Stephan Ripke, Joshua L Roffman, Panos Roussos, Douglas M Ruderfer, Dan Rujescu, Veikko Salomaa, Alan R Sanders, Ulrich Schall, Thomas G Schulze, Sibylle G Schwab, Edward M Scolnick, Rodney J Scott, Larry J Seidman, Pak C Sham, Jianxin Shi, Jeremy M Silverman, Kang Sim, Pamela Sklar, Petr Slominsky, Jordan W Smoller, Hon-Cheong So, Erik Söderman, Chris C A Spencer, David St Clair, Eli A Stahl, Elisabeth Stogmann , Richard E Straub, Eric Strengman, Jana Strohmaier, T Scott Stroup, Mythily Subramaniam, Patrick F Sullivan, Jaana Suvisaari, Dragan M Svrakic, Jin P Szatkiewicz, Srinivas Thirumalai, Draga Toncheva, Paul A Tooney, Sarah Tosato, Peter M Visscher, John Waddington, Dermot Walsh, James TR Walters, Dai Wang, Qiang Wang, Bradley T Webb, Daniel R Weinberger, Mark Weiser, Thomas Werge, Dieter B Wildenauer, Nigel M Williams, Stephanie Williams, Stephanie H Witt, Aaron R Wolen, Emily HM Wong, Brandon K Wormley, Naomi R Wray, Jing Qin Wu, Hualin Simon Xi, Clement C Zai, Xuebin Zheng, Fritz Zimprich.
